# Multiple Sex-Associated Regions and a Putative Sex Chromosome in Zebrafish Revealed by RAD Mapping and Population Genomics

**DOI:** 10.1371/journal.pone.0040701

**Published:** 2012-07-09

**Authors:** Jennifer L. Anderson, Adriana Rodríguez Marí, Ingo Braasch, Angel Amores, Paul Hohenlohe, Peter Batzel, John H. Postlethwait

**Affiliations:** 1 Institute of Neuroscience, University of Oregon, Eugene, Oregon, United States of America; 2 Department of Biological Sciences, University of Idaho, Moscow, Idaho, United States of America; Temasek Life Sciences Laboratory, Singapore

## Abstract

Within vertebrates, major sex determining genes can differ among taxa and even within species. In zebrafish (*Danio rerio*), neither heteromorphic sex chromosomes nor single sex determination genes of large effect, like *Sry* in mammals, have yet been identified. Furthermore, environmental factors can influence zebrafish sex determination. Although progress has been made in understanding zebrafish gonad differentiation (*e.g.* the influence of germ cells on gonad fate), the primary genetic basis of zebrafish sex determination remains poorly understood. To identify genetic loci associated with sex, we analyzed *F_2_* offspring of reciprocal crosses between Oregon *AB and Nadia (NA) wild-type zebrafish stocks. Genome-wide linkage analysis, using more than 5,000 sequence-based polymorphic restriction site associated (RAD-tag) markers and population genomic analysis of more than 30,000 single nucleotide polymorphisms in our *ABxNA crosses revealed a sex-associated locus on the end of the long arm of chr-4 for both cross families, and an additional locus in the middle of chr-3 in one cross family. Additional sequencing showed that two SNPs in *dmrt1* previously suggested to be functional candidates for sex determination in a cross of ABxIndia wild-type zebrafish, are not associated with sex in our AB fish. Our data show that sex determination in zebrafish is polygenic and that different genes may influence sex determination in different strains or that different genes become more important under different environmental conditions. The association of the end of chr-4 with sex is remarkable because, unique in the karyotype, this chromosome arm shares features with known sex chromosomes: it is highly heterochromatic, repetitive, late replicating, and has reduced recombination. Our results reveal that chr-4 has functional and structural properties expected of a sex chromosome.

## Introduction

The process of sex determination specifies an animal’s sex and activates developmental pathways that cause gonads to differentiate into ovaries or testes. The mechanisms of animal sex determination, however, are remarkably diverse. Some animals utilize genetic sex determining mechanisms (GSD) while others rely on environmental cues for sex determination (ESD). GSD systems can be either chromosomal, and involve a master sex-determining gene on a sex chromosome, such as *Sry* in mammals [1], or can be polygenic and involve several genes on multiple chromosomes (*e.g.* [2]). In ESD, the environment plays a decisive role, such as temperature in turtles and alligators [3]–[6]. GSD and ESD can interact in some species; for example, in Japanese medaka (*Oryzias latipes*), which has an XX/XY genetic system, high temperatures can cause female-to-male sex reversal [7]–[13].

Zebrafish (*Danio rerio*) provides a model to understand vertebrate development, physiology, and behavior, but the primary genetic mechanisms that determine zebrafish sex remain elusive. Zebrafish do not have obvious sexually dimorphic chromosomes [14]–[18]. Specific cross designs can yield predictable sex ratios [19]–[22] and genetic loci associated with sex have been identified [23], supporting a role for GSD in this species. Environmental factors such as hypoxia, high temperatures, and crowding can also influence sex ratio in zebrafish [21], [24]–[27], as expected for ESD. Thus, both GSD and ESD appear to function in this species. Because sex ratio can vary widely between crosses, stocks, and generations, sex determination in zebrafish seems to be rather plastic and easily pushed in one direction or the other by genetic or environmental factors [28]. We do not understand, however, the primary genetic mechanisms that underlie these events nor how the environment might perturb them.

Although the genes and primary genetic mechanisms that initiate sex determination in zebrafish remain unknown, downstream genes in the sex determination pathway appear to be highly conserved with other taxa and a general model of sex determination in these fish has been proposed [29]. As juveniles, all zebrafish develop gonads containing immature oocytes [30] and express genes appropriate for the development of both testes (*e.g.*, *amh, sox9a*, *cyp11b*) and ovaries (*cyp19a1a* and *foxl2*) [31]–[34]. Around the fourth week after fertilization, gonads extinguish expression of one or the other set of genes. In some juveniles, oocytes die and the fish become males, but in others, oocytes survive and the fish develop as females [20], [35]. Interestingly, in zebrafish lacking germ cells, somatic cells of the gonad express testis-specific genes, and animals develop exclusively as males [34], [36]–[39]. The presence of germ cells, however, is insufficient to feminize the gonads, suggesting that the presence of oocytes progressing through meiosis is essential to support female development in zebrafish [32], [33], [40], [41]. This shows that post-recombination meiotic zebrafish oocytes provide an as yet unidentified signal to the soma that supports female development [32]. We propose that in zebrafish, genetic and environmental factors act directly or indirectly to regulate the strength of the meiotic oocyte-derived factor [32]. Outstanding questions remain: what are those factors and how do they work?

A better understanding of the molecular genetic basis for sex determination in a species like zebrafish with a delicately balanced sex determining mechanism might lend insight into the increasing rates of human reproductive diseases, such as testicular dysgenesis syndrome (TDS) and polycystic ovary syndrome (PCOS) [42], [43], and provide practical benefits for studies that employ this widely used animal model. To help better understand the genetic mechanisms underlying zebrafish sex determination, we surveyed for sex-associated loci in zebrafish.

In principle, studies comparing the genomes of zebrafish males and females should identify genetic elements that are associated with sex. Quantitative trait locus (QTL) mapping approaches in various fish species have identified chromosome regions containing loci with effects on sex determination [2], [44]–[46]. A recent investigation that mapped sex in a cross between the AB and IN (India) wild-type zebrafish stocks (the ABxIN cross) [23] identified loci on chr-5 and chr-16 [loci here referred to as *sex-associated region chr-5* (*sar5*) and *sar16*]; furthermore, the ABxIN study detected variants in two nearby candidate genes, *dmrt1* in *sar5*, a paralog of which is associated with sex determination in medaka, chickens, and frogs [9], [10], [47], [48], and *cyp21a2* in *sar16* (*steroid 21-hydroxylase*), some mutant alleles of which cause virilization in humans [49]. Additional studies are necessary, however, because sex determination mechanisms can vary even among strains or populations of fish, for example, in poeciliid fish and cichlids [2], [50], and the same may be true in zebrafish.

Here, we analyzed the *F_2_* offspring of reciprocal crosses between Oregon *AB (star AB) and NA (Nadia) wild-type zebrafish stocks to map sex using over 5,000 polymorphic Illumina sequencing based RAD-tag markers [51]–[53] that we genotyped in 231 fish distributed in two families. We observed sex-associated loci on the end of the long arm of chr-4 for both of two *ABxNA families and in the middle of chr-3 for one of the two families, but did not recover the loci found to segregate with sex in the ABxIN cross [23]. Population genomic analyses of genetic differentiation between sexes at more than 30,000 single nucleotide polymorphisms (SNPs) per cross family confirmed the mapping results. Furthermore, two SNPs in *dmrt1* previously proposed as functional candidates [23] failed to associate with sex in our fish. We conclude that sex determination in zebrafish is likely to be polygenic and genes that influence sex development may be strain or environment specific. Furthermore, the sex-associated locus on chr-4 (*sar4*) occupies the only chromosome arm in the zebrafish karyotype that is late-replicating, repeat-rich, and heterochromatic [14], [16], [18], properties that characterize mature sex chromosomes in many other species. This result raises the possibility that chr-4 is a sex chromosome in zebrafish.

## Materials and Methods

### Ethics Statement

The University of Oregon Institutional Animal Care and Use Committee approved all animal work (Animal Welfare Assurance Number A-3009-01, IACUC protocol # 11-07).

### Stocks and Cross Design

Nadia and *AB (star AB) fish were obtained from the zebrafish facility at the University of Oregon. *AB stocks were derived in 1991–1992 from George Streisinger’s original AB stocks [54] that had been maintained at the University of Oregon since the 1970s (http://zfin.org/action/genotype/genotype-detail?zdbID=ZDB-FISH-960809-7). Nadia stocks originated from wild populations of zebrafish north of Kolkata (Calcutta), India, and have been in culture at the University of Oregon since 1999 (http://zfin.org/action/genotype/genotype-detail?zdbID=ZDB-GENO-030115-2). All fish were reared as previously described [55]. The University of Oregon Institutional Animal Care and Use Committee approved all animal work (Animal Welfare Assurance Number A-3009-01, IACUC protocol # 11-07).

Reciprocal single-pair matings of *AB and Nadia fish generated *F_1_* individuals. Full-sib single-pair matings of *F_1_* fish produced *F_2_* individuals used for mapping. Two *F_2_* families were used, one from the initial *F_0_* cross between a Nadia female and *AB male (Family A) and the other from the reciprocal *F_0_* cross between a *AB female and a Nadia male (Family B). Family A consisted of 86 female and 103 male *F_2_* siblings. Family B consisted of 56 female and 96 male *F_2_* siblings. The sex of *F_0_* and *F_1_* individuals was assessed by the examination of sexually dimorphic external characters and the types of gametes each fish produced. The sex of *F_2_* individuals was assessed by external characters and confirmed by the dissection and direct observation of the gonads, classifying them as either ovaries or testes. Fish that could not be confidently sexed after gonad dissection were excluded from further analysis.

*RAD Genotyping–*RAD (Restriction site Associated DNA) libraries were created as previously described [51], [52]. In brief, genomic DNA was extracted from muscle using a Qiagen DNeasy Blood and Tissue Kit and digested with high-fidelity *Sbf*I restriction enzyme (New England Biolabs). Barcoded adapters were ligated to each sample. Barcodes were 6 nucleotides (nt) long and differed from each other by at least 2 nt to facilitate unambiguous identification of each sample following sequencing. Samples were pooled, sheared, and the 200–500 base pair (bp) size fraction purified from an agarose gel after electrophoresis. Following ligation of a second adaptor, fragments containing both adapters were PCR amplified (12 cycles) and the 200–500 bp size fraction isolated as above. RAD libraries were sequenced on an Illumina HiSeq 2000 using 100 bp single-end reads. We RAD-sequenced all *F_0_* and *F_1_* fish used to produce Families A and B, 167 *F_2_* siblings from Family A, and 128 *F_2_* siblings from Family B. Sequences are available online at the NCBI Sequence Read Archive (http://trace.ncbi.nlm.nih.gov/Traces/sra/sra.cgi; accession number SRA049620.1).

Sequence data were processed using the *process_radtags* component of the *Stacks* pipeline (http://creskolab.uoregon.edu/stacks/) [56], which removes reads of low quality and bins data by barcode. Sequences were then aligned to the zebrafish genome (Zv9) using Bowtie 0.12.5 [57]. Sequences that aligned to more than one place in the genome or that aligned to unassembled scaffolds were excluded from further analysis. RAD-tag markers were identified in the *F_1_* parental fish from each cross and scored in *F_2_* individuals in *Stacks*. Conservatively, a minimum read-depth of 20× coverage per marker per individual was required for a sequence to be identified as a RAD-tag in *Stacks*. Genotypes, which were formatted for map type CP (outbred full-sib family), were then exported from *Stacks* and used for genetic map construction.

### Genetic Map Construction, Linkage Analysis, and Recombination Rates

Genetic maps for Family A and Family B were generated in JoinMap 4.1 (Wageningen, The Netherlands), using the Kosambi mapping function and a multipoint maximum likelihood mapping algorithm for cross type CP [58]. Maternal and paternal markers were used to construct separate male- and female-specific linkage maps for each chromosome to facilitate identification of improbable double recombination events and incorrect marker order. Genotypes associated with double recombination events, as identified by JoinMap, were verified or corrected in *Stacks* (as in [52]). Marker order was initially based on positional data from Zv9 and manually refined. New male- and female-specific maps were generated using corrected genotypes and refined marker orders and analysis was reiterated until suspicious genotypes and double recombination events were minimized. Consensus maps were then generated for each chromosome. Markers with significant segregation distortion (P<0.01), unlinked at LOD<6, or with excessive missing data were excluded from analysis. Individuals with read depth lower than 20 reads for more than 20% of the markers for Family A and more than 30% for Family B were also excluded. Residual improbable double recombination events were identified and treated as above. Genotype data were exported from JoinMap and recoded for analysis as a phase-known 4-way cross in R/qtl using a custom python script (Supporting Text S1). Linkage analysis to identify loci associated with sex was performed in R/qtl [59] using interval mapping and a model appropriate for the analysis of a binary trait. Statistical significance for linkage to sex was determined using a permutation test (10,000 permutations).

We calculated sex-specific recombination rates in Family A from marker positions in the final male- and female-specific maps (cM, above) and marker position in Zv9 (Mb) using a sliding window of 3 markers (moving 1 marker per estimate).

In previous work, numbers were assigned to zebrafish linkage groups (LGs) based on genetic length [60], [61]; these LGs are called ‘chromosomes’ in the Zv9 version of the zebrafish genome at Ensembl, and we abbreviate them here as ‘chr’. Subsequently, LGs were assigned to physical chromosomes [18] that cytogeneticists had previously numbered based on physical length [15], [16], [62]–[64]. In this paper, we use the designation ‘chr’ to mean ‘Linkage Group’ rather than cytogenetically identified physical chromosomes, in agreement with zebrafish nomenclature conventions. Thus, chr-4 herein (Linkage Group 4) is cytogenetic Chromosome-3 and chr-3 (Linkage Group 3) is cytogenetic Chromosome-4 [18].

### SNP Genome Scan

We also tested the association of sex with genotypes at individual single nucleotide polymorphisms (SNPs) across the genome in the *F_2_* individuals. Starting with the sequence data aligned to the reference genome with Bowtie (from above), we assigned a diploid genotype at each nucleotide position in each individual using a modification of the maximum likelihood genotyping method of [53]. This method assigns a diploid genotype if a likelihood ratio test between the two most likely genotypes is significant (here at á = 0.01), and does not assign a genotype if the test is not significant, based on a multinomial sampling model of read counts for alternative nucleotides. This method implicitly results in a minimum threshold for sequencing depth, depending on the ratio of read counts for alternative nucleotides, because low-coverage sites will not result in a significant likelihood ratio test. Thus we did not explicitly filter for sequencing depth in this analysis. We modified this method to include a bounded uniform prior distribution on the per-nucleotide sequencing error rate å from 0.001 to 0.1; that is, the likelihood for each possible genotype was calculated as the maximum only within this range of å (see equation 1 in [53]). This approach reduces the error rate of assigning a homozygous genotype when the true genotype is heterozygous but with uneven or biased read counts, as occurs in Illumina-based RAD sequencing (Hohenlohe, unpublished data). Within each family, we filtered out SNPs that were genotyped at fewer than 20 male or 20 female individuals. We then conducted a G-test for significant genetic differentiation between males and females at each SNP [65], and we calculated the false discovery rate within each family using the algorithm of Benjamini & Hochberg [66].

### Partial Sequencing of *dmrt1*

The partial sequencing of *dmrt1* from one male and one female in the ABxIN cross identified two potentially functional sex-specific SNPs: dbSNP ss184964113 and ss184964140 [23]. To determine whether these SNPs are associated with sex in our AB fish stocks, we sequenced from 12 males and 12 females, 628 bp of *dmrt1*, including exon-5 and part of the 3¢UTR and both potentially functional sex-specific SNPs using forward primer 5¢CCCAAACCAGATTACGCTCTGGCA and reverse primer 5¢TGCATCATGAAGGTCGCGGG. Heterozygous base positions were identified by Geneious v5.5 [67] and verified by manual inspection.

### Genomic Analysis of chr-4

To address the question of whether chr-4 has, and is unique in having, characteristics of a sex chromosome, we downloaded the locations of genes in the zebrafish genome assembly Zv9 from Ensembl65 using Biomart (http://www.ensembl.org/biomart/martview/). Protein coding genes were analyzed according to their Ensembl entries. Repeat elements for Zv9 were downloaded from the Table Browser of the UCSC Genome Browser (http://genome.ucsc.edu/cgi-bin/hgTables?org=zebrafish). Genomic distribution of genes and other elements was visualized using the Integrative Genomics Viewer (IGV 2.0.34) [68].

Chromosome spreads were obtained from fibroblast tissue cultures from caudal fin clips [16], [69], from 4 male and 4 female individuals. For replication banding, 5-bromodeoxyuridine (BrdU) (30 µg/ml final concentration) was added to the cultures 6–8 hours before harvesting the cells. Colchicine (0.02 µg/ml final concentration) was added 1–2 hours before harvesting the cells. Collected cells were treated in a hypotonic solution of 0.075 M KCl for 20 minutes at room temperature. The cell suspension was fixed by four washes in 3∶1 methanol-acetic acid. Cells were spread onto cleaned slides and air-dried. For replication banding, we immersed dry slides for 30 min in Hoechst 33258 (1 µg/ml), rinsed in distilled water, and covered them with a thin layer of 2× SSC before exposure to a 15 W Sylvania G15T8 germicidal lamp at a distance of 10 cm for 60 min. After incubating slides in 0.5×SSC at 60°C for 60 min, we rinsed slides in distilled water and stained in 4% Giemsa for 20 min. We investigated more than 10 different metaphases per individual.

## Results

### Sequences, SNPs, and Genetic Maps

To identify loci linked to sex, we constructed *F_2_* mapping populations from two distantly related zebrafish strains. We sequenced *Sbf*I-associated RAD-tags in 167 offspring from Family A (*AB grandsire and Nadia granddam) and 128 offspring from Family B (the reciprocal cross, Nadia grandsire and *AB granddam) (Table 1). RAD-tag sequencing identified about 46,000 tags, each 100 nucleotides long, associated with *Sbf*I cut sites genome wide (Table 1). Approximately 37% of all sequenced tags contained at least one SNP in the *F_1_* parents for each cross; of these, 5180 tags were genotyped in a minimum of 90% of individuals in Family A. In Family B, 6357 tags were genotyped in at least 70% of the *F_2_* offspring. These polymorphic markers were exported from *Stacks* for use in genetic map construction. Following the exclusion of markers and individuals due to missing data and segregation distortion criteria (see methods), genetic maps of length 2346 cM (centiMorgans) for Family A (145 individuals) and 2333 cM for Family B (86 individuals) were constructed based on 4796 or 5794 markers respectively (Table 1, Figure S1). The genetic and physical positions of mapped markers can be found in Tables S1 and S2. Overall, fewer than 10% of the polymorphic markers were excluded during genetic map construction. In Family B, nearly all markers on chromosome 14 exhibited significant segregation distortion but were retained for analysis.

**Table 1:** Characteristics and outcomes of *ABxNA crosses, RAD-tag sequencing, and genetic mapping.

	Family A	Family B
Grandsire	*AB	Nadia
Sex ratio (male:female)	1.2∶1	1.7∶1
# Mapped individuals [total (male, female)]	145 (82, 63)	86 (48, 38)
Sequenced RAD tags	46,425	46,313
Polymorphic tags	5180	6357
Mapped markers	4796	5794
Genetic map size (cM)	2346	2333

### Linkage Analysis

**Figure 1:**
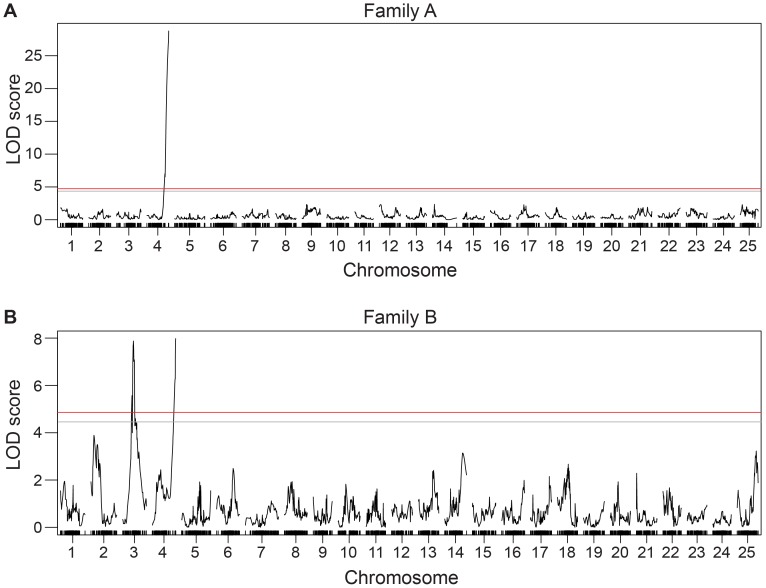
Mapping results for the *F_2_* of reciprocal crosses between *AB and NA wild type stocks. (A) Family A, *AB grandsire. (B) Family B, NA grandsire. Sex is associated with a locus on chr-4 in both families, and a locus on chr-3 in Family B. LOD scores are plotted in order of marker position in cM. Significance thresholds were determined by permutation testing and are indicated by horizontal lines (red = 5%, gray = 10%).

Linkage analysis identified one sex-associated region on chr-4 in both cross families and an additional locus on chr-3 linked to sex in Family B (Figure 1). Hereafter, we refer to the chr-4 locus as *sar4* for *sex-associated region-4*, and the chr-3 locus as *sar3*. In Family A, *sar4* peaks at 92 cM [LOD 28.78, marker ID29464 near Zv9 physical position 4∶61,176,889 bp (base pairs); 1.5 LOD drop interval from 91.0 cM (marker ID29449 near 4∶60,837,953 bp) to the end of chr-4 at 62,094,675 bp]. In Family-B, the *sar4* peak is at 98.4 cM [LOD 7.98, marker ID32525 near physical position 4∶61,422,807 bp, 1.5 LOD drop interval from 96.7 cM (marker ID32485 near physical location 4∶60,837,953 bp) to the end of chr-4 at 4∶62,094,675 bp]. In Family B, *sar3* resides at 45.3 cM [LOD 7.87, marker ID29552 near physical position 3∶20,676,406 bp; 1.5 LOD drop interval from 44.1 cM to 49.9 cM (physical positions marker ID29370 near physical position 3∶15,234,176 to marker ID29830 at 3∶29,193,306 bp)]. These peaks exceed genome-wide significance for linkage to sex in their respective crosses (Figure 1).

**Figure 2:**
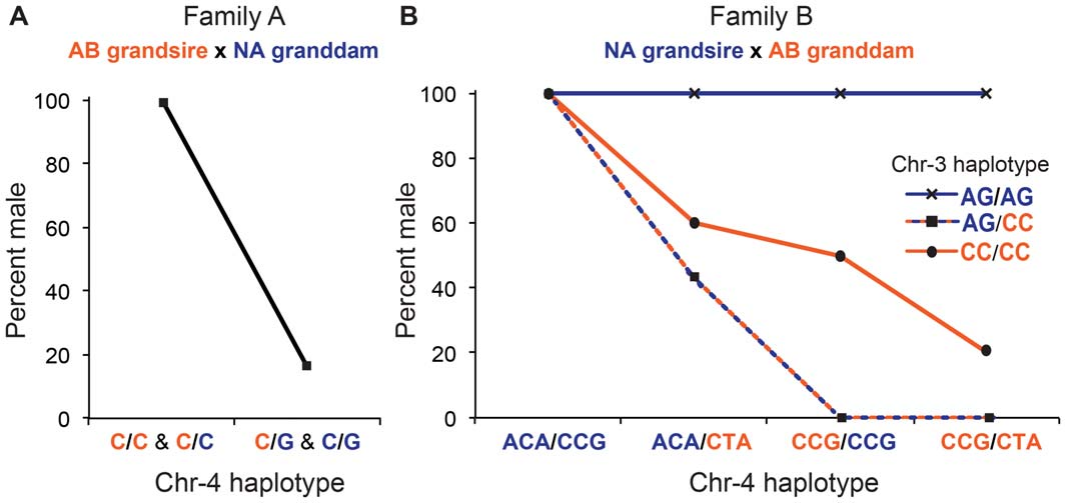
Relationships between sex phenotype and haplotype on chr-3 and -4. (A) In Family A, all individuals homozygous for the C allele at Marker ID29464 chr-4∶61,176,889 bp were male and 84% of individuals heterozygous at this marker were female. (B) In Family B, loci on chr-3 and -4 appear to interact to influence sex (marker ID32525 chr-4∶61,422,807bp; marker ID29552 chr-3∶20,676,406bp). Individuals homozygous for grandsire-derived alleles at the chr-4 locus developed as males independent of their chr-3 genotype and animals homozygous for granddam-derived alleles were all or nearly all female without regard to their chr-3 genotype. On the other hand, individuals that were homozygous for the grandsire-derived alleles at the chr-3 locus were male independent of their genotype at the chr-4 locus.

The marker at the peak of *sar4* in Family A contains one SNP (C/G). All 70 individuals homozygous for the C allele at this SNP were male (one or both C alleles inherited from the homozygous C/C grandsire and the other C inherited from the heterozygous G/C granddam), whereas 84% (63/75) of C/G heterozygotes were female, the G allele having passed from the NA granddam through the heterozygous C/G dam and the C inherited either from the grandsire or the granddam (Figure 2A, Table S3). While it might be tempting to interpret this relationship between haplotype and phenotype as evidence of a ZW/ZZ mode of sex determination, it must be noted that not all adjacent markers, which were also statistically associated with sex in this analysis, support this interpretation. Thus, until the causative genetic factor in *sar4* is identified or more closely mapped, it is premature to ascribe an XX/XY or ZW/ZZ role to *sar4* (likewise for *sar3* below).

In Family B, relationships between haplotype and sex were more complex: some combinations of haplotypes on chr-3 and chr-4 were found in both males and females but others were found exclusively in either males or females (Figure 2B, Table S4). All 16 individuals with the grandsire-derived ACA/CCG alleles for *sar4* were male independent of their genotype at *sar3*, and all 18 fish with the granddam-derived genotype CCG/CTA or homozygous for CCG (one copy originating from each grandparent), were female if they were heterozygous AG/CC at *sar3*. These results show that multiple genes can influence sex determination in zebrafish and that genes affecting sex determination may differ among strains.

### SNP Genome Scan

**Figure 3:**
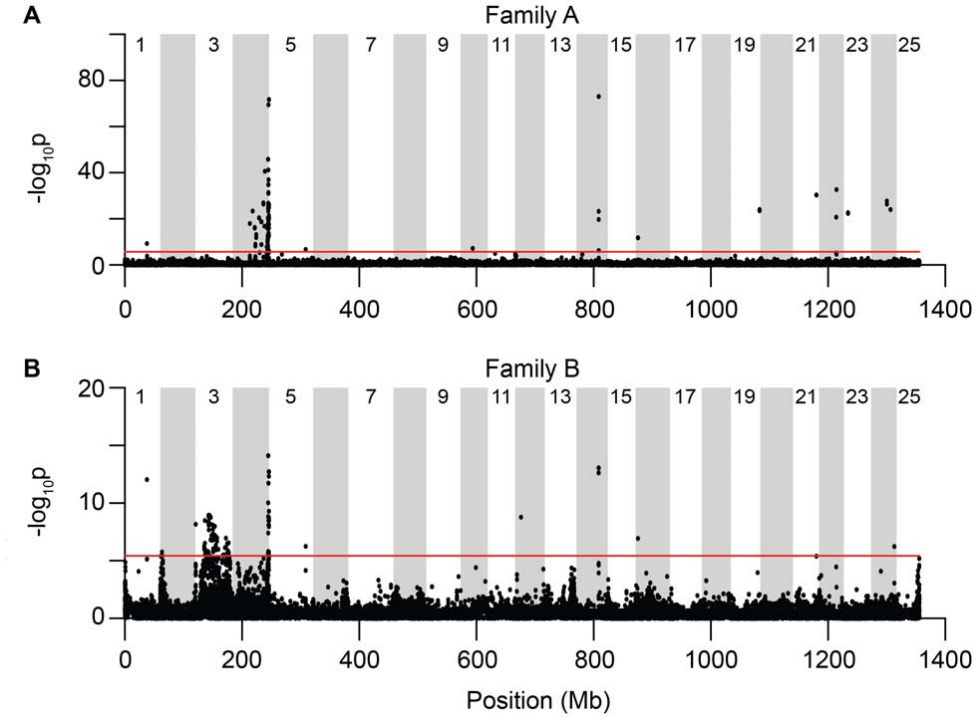
Genome wide differentiation between males and females. (A) Family A. (B) Family B. Individual SNPs were tested for their non-random association with male or female phenotypes. Graphs plot the -log_10_ *p*-value of a G-test across the genome and show peaks at the *sar4* and *sar3* locations. Red lines show the 0.1 percent false discovery rate within each family. Peaks in chr-14 are in genes that are members of a major multigene family in *sar4*, and may be mis-localized in the current genome assembly.

A total of 36,860 SNPs in Family A and 36,370 SNPs in Family B were genotyped in 20 or more individuals of each sex in each family. We tested association of genotype with sex at each of these SNPs using a G-test [65], corrected for false discovery rate within each family [66]. Information about the significant sex-associated SNPs can be found in Tables S5 and S6. Individual SNPs significantly associated with sex (Figure 3) closely matched genomic regions *sar3* and *sar4* identified in the linkage analysis (Figure 1). In Family A, SNPs on chr-4 at physical locations 62,076,809 bp and 62,076,876 bp exhibited the strongest association; additional highly significant SNPs (−log_10_ *p*>40) clustered between 60,613,763 bp and the end of chr-4. In Family B, highly significant SNPs (−log_10_ *p*>10) occurred between physical positions on chr-4 at 60,762,591 bp and 61,934,267 bp. On chr-3, an additional broad cluster of significant SNPs in Family B matched the peak observed in the linkage analysis. In addition, a small cluster of SNPs representing just two RAD-tags in both families was significantly associated with sex in a 24 kb region on chr-14, between physical locations 37,844,124 bp and 37,868,461 bp. These two markers fell in two adjacent predicted genes (CABZ01075275.1 and CABZ01075273.1) that Ensembl scores as having 226 paralogs, most of which are on the long arm of chr-4, many in *sar4*; these markers were excluded from the linkage analysis above because they were genotyped to criterion depth in too few animals. About 40% of BAC clones that uniquely hybridize to the long (q) arm of chr-4 (chr-4q) were assembled onto chr-14 in the Zv6 version of the zebrafish genome [70]; in Zv9, most of these BAC end sequences were assembled onto chr-4q (data not shown). Therefore, it is possible that the sex associated chr-14 SNPs we identify here may also belong on chr-4q like the other originally misassembled BACs.

### Partial Sequencing of *dmrt1*

In the ABxIN cross, *dmrt1* from one male and one female fish, each homozygous for alternative male- and female-associated alleles, was resequenced, and found to differ by 162 SNPs, 28 indels, and two simple tandem repeats [23]. Two of these SNPs within *dmrt1* were suggested to be functional candidates [23]. To test whether these SNPs were associated with sex in our AB fish, we sequenced a 628 bp region of *dmrt1* in 12 female and 12 male AB fish. All 24 fish in our study, including both males and females, were homozygous for C at ss184964113 (a C/A polymorphism causing a nonsynonymous (D259E) substitution in Dmrt1) [23]. At ss184964140, an A/G SNP in what is thought to be the *cis*-regulatory motif of the 3¢UTR [71], [72], our 24 AB fish were either homozygous for A, or A/G heterozygotes, but the genotype at this SNP was not associated with sex (Fisher’s exact test P = 0.21). We observed polymorphisms at 7 additional positions (Text S2), but none were associated with sex. Rather, both males and females were either homozygous throughout this region, or heterozygous at all eight polymorphic sites. We conclude that the candidate functional SNPs in the ABxIN cross [23] are not associated with sex in our AB stocks and thus may not function as sex determinants in all strains of zebrafish.

### Recombination Across the Genome

**Figure 4:**
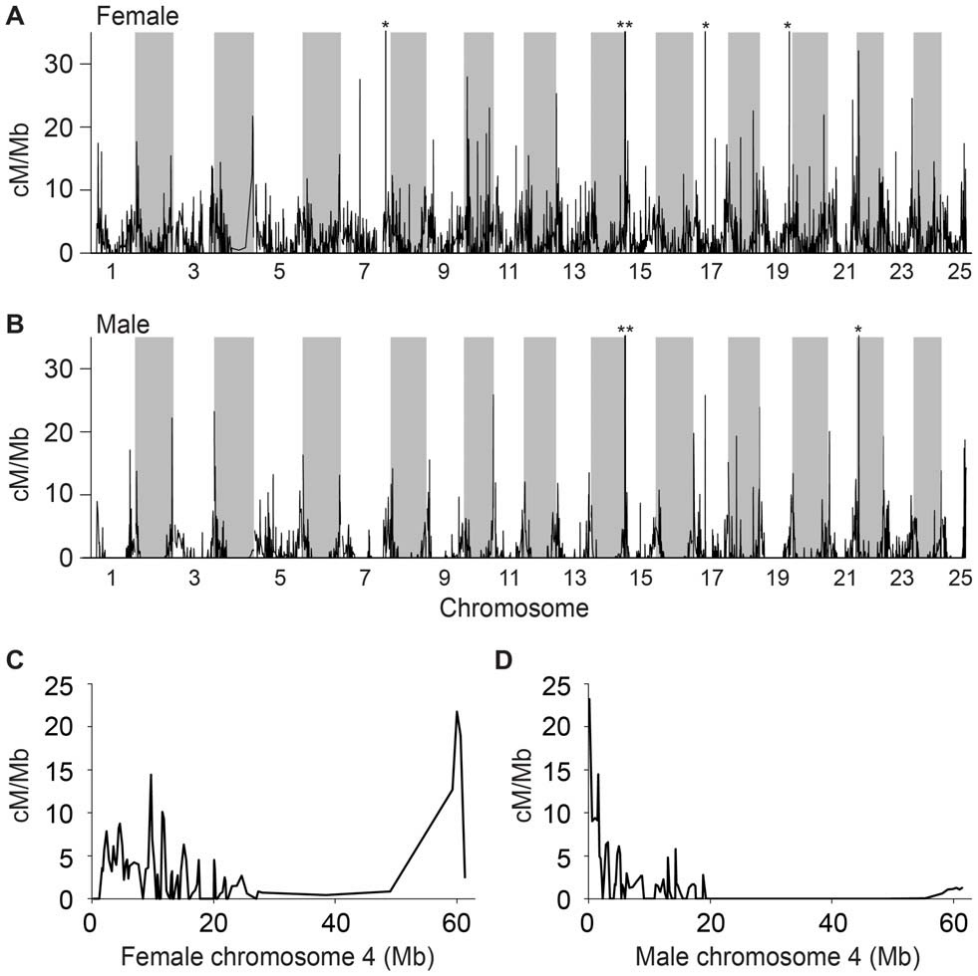
Sex-specific recombination rates across the genome. (A) Female-specific recombination genome-wide. (B) Male-specific recombination genome-wide. (C) Female-specific recombination across chr-4. (D) Male-specific recombination across chr-4. Recombination rates were measured in a sliding window comparing the map distance between markers to the genomic location on the Zv9 assembly of the zebrafish genome. Recombination is generally elevated at telomeres but is reduced in males at *sar4*. For ease of visualization, recombination rates of excess of 35 cM/Mb at the top of the graph are indicated by *. In the female, these rates equal 88, 49, 67, 52, and 108 cM/Mb from right to left. In the male, these rates equal 94, 50, and 39 cM/Mb.

A major feature of sex determining chromosomal regions is the suppression of recombination [73]–[76]. To investigate genome-wide recombination rates, we performed a sliding window analysis of the ratio of genetic map distance in cM per Mb of the Zv9 assembly of the zebrafish genome for both the male genetic map and the female map. Results showed first, that in general, the recombination rate is reduced in males compared to females (Figure 4A, B), thus corroborating earlier results for a genetic map based on male meiosis [77]. Second, results showed that in general, for both males and females, recombination rates tended to be higher towards the telomeres on most chromosomes, also confirming prior findings for a sex-averaged map [23]. Third, the comparison of the rates of recombination across chr-4 in males and females showed that recombination was greatly reduced in males around the sex-associated region at distal chr-4q (Figure 4C, D). This type of recombination suppression is expected of chromosome regions containing sex-determining factors [73]–[76].

### The Physical Nature of chr-4q

**Figure 5:**
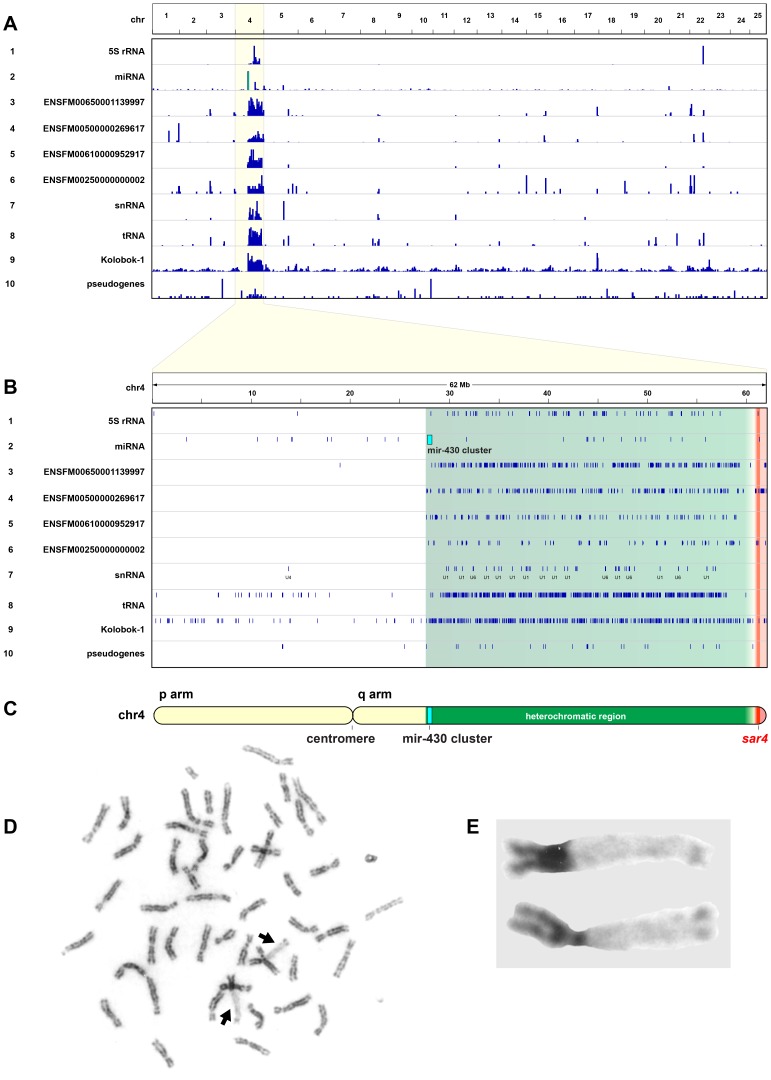
Repetitive nature of the hetetochromatic region of chr-4 compared to the whole zebrafish genome. (A) Genome wide distribution (chr-1 to −25 across the top of the figure) of genetic elements enriched in the heterochromatic region of chr-4. Rows listed top to bottom: 5S RNA motif, micro RNAs (miRNAs), four different Ensembl multigene families (ENSFM), small nuclear RNAs (snRNAs), transfer RNAs (tRNAs), Kolobok-1 transposon, and pseudogenes. See Table 2 for specifics on these elements. (B) Within chr-4 (position in Mb listed across the top), enriched elements are found in the hetrochromatic region (green) delineated by the *mir-430* cluster (light blue) at the centromeric side and *sar-4* (red) near the telomere of the p arm. (C) Schematic of chr-4 with locations of the centromere, heterochromatic region and *sar-4*. (D) Replication banding of a cell cultured from a female zebrafish showing the late replication of chr-4q (arrows), unique among the 50 zebrafish chromosome arms. (E) Enlargement of the chr-4 pair; note heterochromatic chr-4q.

To investigate whether chr-4 is unique in the genome in possessing features commonly found in sex chromosomes, we compared the distribution of various genetic elements across the genome (Figure 5, Table 2). First, chr-4q is the only arm in the zebrafish genome that is heterochromatic. Chr-4 (as designated at Ensembl and originally defined as Linkage Group-4 [60], [61]) is the third largest cytogenetic chromosome in zebrafish [18] and is used as a standard for cytogenetic comparisons because of its unique heterochromatic long (right) arm (chr-4q) [14]–[16], [64]. The heterochromatic region on chr-4 in genome assembly Zv9 begins at about 28 Mb and contains *sar4* at about 61–62 Mb. This region comprises about 2.26% of the entire genome assembly. Zebrafish centromeres have been mapped [61], [78]–[80], showing that the microsatellite marker Z10280 (GenBank accession G39774) lies near the centromere of chr-4 at 20.3 Mb in Zv9 (Figure 5B). Some of the repetitive elements of the heterochromatic region mentioned below extend into the euchromatic region at the end of chr-4q.

**Table 2:** Repetitive genetic elements in the heterochromatic region of chr-4.

Genetic Element	Ensembl Family Name(molecular function)	# in Genome (Zv9)	expected in hetero-chromatic region^1^	# in hetero-chromatic region	% in hetero-chromatic region
ENSFM00650001139997	– (zinc finger)	436	9.9	261	59.9
ENSFM00500000269617	–	417	9.4	185	44.4
ENSFM00610000952917	–	147	3.3	97	66.0
ENSFM00250000000002	– (zinc finger)	189	4.3	54	28.6
ENSFM00500000273896	ms4a17a	16	0.4	15	93.8
ENSFM00600000925039	–	12	0.3	11	91.7
ENSFM00600000923389	–	13	0.3	8	61.5
miRNA	*mir-430*	56	1.3	55	98.2
snRNA	U6	706	16	460	65.2
	U1	382	8.6	224	58.6
tRNA		22,275	503	10,396	46.7
Kolobok 1		4,445	100	635	14.3
Pseudogenes		200	4.5	24	12.0

1 Expected number in the heterochromatic region of chr-4 (covering 2.26% of the Zv9 genome assembly) if elements were uniformly distributed in the genome.

Chr-4q has accumulated numerous special genetic elements, including 5S RNA motifs, miRNA repeats (especially *mir-430* see below), a number of repetitive gene families, snRNA genes, tRNA genes, the transposable element Kolobok-1, and pseudogenes. Cytogenetic studies showed that 5S rRNA probes hybridize to chr-4q [62]–[64] and this is confirmed by the distribution of the 5s rRNA sequence motif in Zv9, with a strong enrichment on chr-4q (Table 2, Figure 5A, B row 1). The centromere-proximal portion of the heterochromatic region includes a cluster of 55 members of the miRNA family *mir-430* at about 28.0 Mb. Only one *mir-430* gene is found outside this cluster, on chr-10 (Table 2, Figure 5A, B, row 2).

Chr-4q is enriched for multiple copies of genes belonging to several specific Ensembl protein families. Figure 5 A and B (rows 3–6) illustrate four gene families that have 189 to 436 copies spread throughout the genome, with substantial concentrations (28–65% of the copies) located in the heterochromatic region of chr-4q (Table 2). These include a zinc-finger fragment family and others of uncharacterized nature. Notably, chr-4q is enriched in additional multigene families with lower copy numbers (Table 2), such that these families are found nearly exclusively in the heterochromatic region of chr-4q.

Chr-4q contains a number of additional repetitive elements. Small nuclear RNAs (snRNAs) are enriched on chr-4 (Table 2, Figure 5A, B row 7). This enrichment is due specifically to the abundance of snRNAs U6 and U1. Nearly half of all tRNAs in the zebrafish genome are clustered in the heterochromatic region of chr-4q. (Table 2, Figure 5A, B row 8). While chr-4q is not enriched for transposons in general, it is enriched for copies of the transposon Kolobok 1, a member of the autonomous Kolobok transposon family (Figure 5A, B and S2) [81]. Other members of the Kolobok family are not highly represented on chr-4 (Figure S2), but 14% of all Kolobok 1 copies in zebrafish are found within the approximately 34 Mb long heterochromatic region of chr-4q (Table 2).

Many sex chromosomes are enriched in pseudogenes. In the zebrafish genome, 12% of all Ensembl annotated pseudogenes reside in the heterochromatic region of chr-4q (Table 2, Figure 5A,B row 10).

The X-chromosomes of mammalian female somatic cells are late replicating [82]. The incubation of zebrafish cells with bromodeoxyuridine (BrdU) revealed that chr-4q is also late replicating in zebrafish (Figure 5C,D). Results were consistent in all metaphases analyzed, and no major differences were observed between the male and female karyotypes. Figure 5 (C and D) illustrates a typical karyotype from a female individual.

We conclude that chr-4 possesses a number of properties generally associated with mature sex chromosomes, not the least of which is a sex-associated locus.

## Discussion

The primary genetic mechanisms that control gonad fate and determine sex in zebrafish remain poorly understood. This gap in our understanding persists despite several decades of intense investigation on zebrafish development and genetics. Developmental studies based on candidate genes show that the zebrafish sex determination pathway shares many genes in common with gonad developmental pathways in other vertebrate taxa. These approaches, however, have not yet identified the master genetic factors that initiate sex determination. To advance our understanding of sex determination in zebrafish, it is necessary to identify these factors. To help identify genes associated with sex in zebrafish, we genotyped 231 *F_2_* offspring from reciprocal crosses between NA and *AB wild-type strains at more than 46,000 *Sbf*I associated RAD-tags distributed throughout the genome, on average one every 37 kb and analyzed genotypes using both quantitative genetic and population genomic methods. Analyses converged on the identification of two genomic regions linked to sex. One of those regions, *sar4*, lies on the end of chr-4q and was linked to sex in both of our cross families (Figure 1, 3). The other sex-associated region, *sar3*, was in the middle of chr-3 and was identified in only one of the crosses (Figure 1, 3). These results suggest that sex determination in zebrafish is polygenic and that different genes may influence sex development in different strains and/or under different environmental conditions. Furthermore, the association of the end of chr-4q with sex in both cross families is of particular interest because chr-4 shares many physical features with known sex chromosomes.

### Multiple Loci Influence Sex Determination in Zebrafish

**Figure 6:**
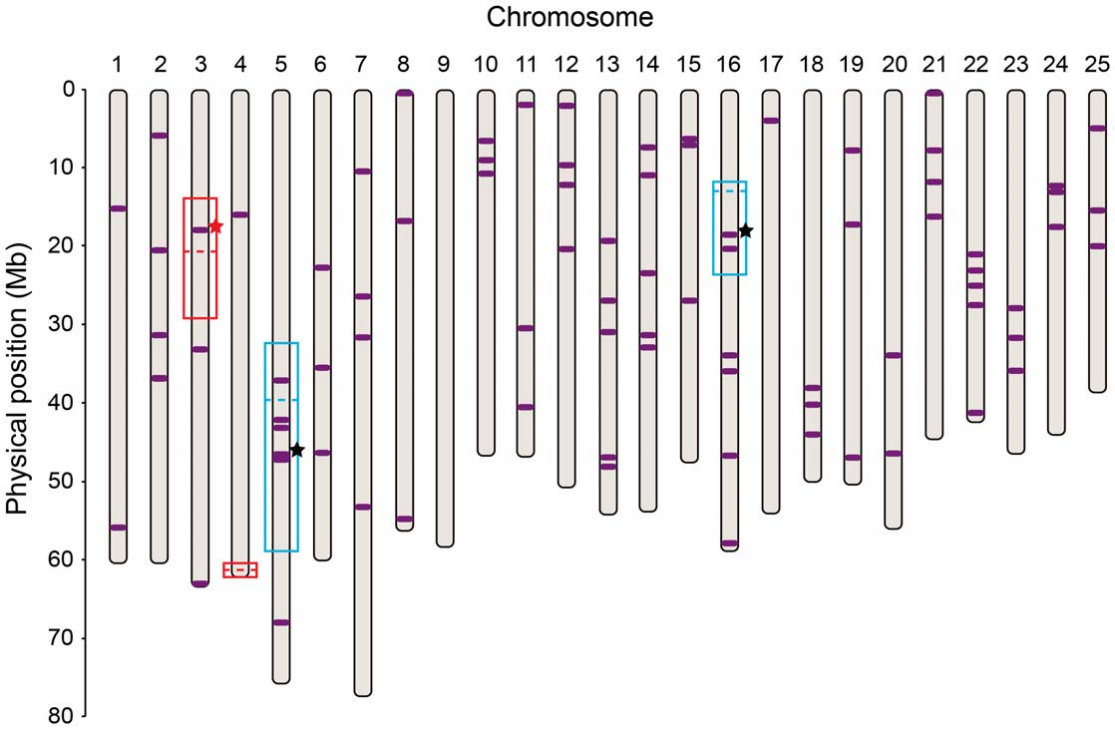
Genomic locations of candidate sex genes and the locations of mapped sex-associated regions. The physical positions of genes with a potential influence on sex determination in zebrafish are marked in purple (see for names and locations the full list in Table S7). Red boxes indicate the 1.5 LOD drop intervals for *sar3* and *sar4* identified here in the *ABxNA cross and blue rectangles mark the 1.5 LOD droop intervals for *sar5* and *sar16* in the ABxIN cross [23]. Dashed lines mark peaks of sex-associated loci identified herein (red) and by Bradley *et al.* [23] (blue). Black stars indicate genes identified as candidates for functional sex determination genes in zebrafish by Bradley *et al.* [23]. The red star indicates *hsd17b1*. Upper and lower bounds of the surrounding boxes represent the 1.5 LOD drop intervals for each peak.

In some taxa, including mammals and medaka, a single gene of large effect controls sex determination (*Sry* and *Dmrt1by* respectively). We found, however, that in one of the *F_2_* families of our *ABxNA cross, more than one locus was associated with sex (Figure 1, 3, 6). The sex associated loci in the ABxIN cross ([23] loci referred to herein as *sar5* and *sar16*), and classical breeding experiments in zebrafish [22] further support the polygenic nature of zebrafish sex determination. Polygenic sex determination mechanisms have been observed in several fish. For example, gilthead sea bream (*Sparus aurata*), red top zebra cichlid (*Metriaclima pyrsonotus*), and platyfish (*Xiphophorus maculatus*) all have polygenic sex determination systems [2], [46], [83].

The genetic basis of sex determination in zebrafish appears to differ among strains or environmental conditions. We identified two loci associated with sex in zebrafish. The *sar4* locus was associated with sex in both Family A (*AB grandsire) and Family B (NA grandsire). The *sar3* locus, however, was sex-associated only in Family B. Two additional sex-associated loci were identified in the ABxIN cross (IN grandsire) [23]. The three crosses studied to date differ in that grandsires (*AB, NA, IN) and granddams (NA, AB, and its derivative *AB, see methods) from different strains were used in each. Thus, one explanation for the observation of different sex-associated loci in these crosses is that zebrafish harbor intraspecific variation for genes influencing sex determination. Recent population genetic analyses show that wild populations of zebrafish are variable and divergent and that the AB laboratory strain differs markedly from outbred wild populations [84]–[86]. While both IN and NA originate from India, the IN strain originates from Darjeeling and the NA strain comes from Nadia north of Kolkata; thus, it is possible that these strains are genetically divergent. The AB strains used in the two different laboratories had a common origin in the initial AB strains established in Oregon by Streisinger and collaborators [54] and are separated by only a few tens of generations. Different strategies for stock maintenance or genetic drift may have left the two derivative strains with different sex determination systems. It has recently been shown that different zebrafish mating pairs can repeatedly produce widely different sex ratios, from about 5% to about 97% males, consistent with variation in sex determining genes [22].

An alternative explanation for finding different sex associated loci in different experiments is that the environmental conditions under which the ABxIN and *ABxNA crosses were grown differed in subtle, unknown ways important for sex determination and it could be that different sex determining genes become more important in different environments. Recent experiments [27], [87] show that high temperature alters sex ratios in zebrafish in a genotype-specific fashion.

Precedents exist for strain-specific sex determination mechanisms in other fish. Most guppy families (*Poecilia reticulata*) have an XX/XY mechanism, but autosomal factors in some families can cause XY- or XX-sex reversal [88], [89]; the platyfish (*X. maculatus*) has three female genotypes (XX, XW, or ZW) and two male genotypes (YY or XY) [83]; and the red top zebra cichlid (*M. pyrsonotus*) can have XY and ZW sex determination systems that both segregate even within the same family [2]. Furthermore, closely related species of stickleback, medaka, salmonids, and cichlids all appear to have evolved sex chromosomes involving different linkage groups [90]–[93].

### Sex-associated Regions

Four sex-associated regions have now been identified in zebrafish. Three of these regions (*sar3*, *sar5*, *sar16*) contain genes known to influence sex development, genes we might *a priori* consider to be candidate genes for sex determination (Figure 6, Table S7). Bradley *et al.* [23] considered two candidate genes, *dmrt1* (*sar5*), and *cyp21a2* (*sar16*) in their study. They identified compelling, sex-specific mutations in these genes that could suggest mechanisms for sex determination in zebrafish; to our knowledge, however, no reports yet confirm the causative roles of these genes and polymorphisms in sex determination in zebrafish. Interestingly, the putative functional SNPs in *dmrt1* are not associated with sex in our stocks of AB wild-type fish; this result could be due to strain-specific differences in sex determination in these fish. Regardless, additional strong candidate genes reside within *sar5* and *sar16* that could underlie sex determination in the ABxIN cross, including *fancg*, which lies closer to the peak of *sar5* than *dmrt1* (Figure 6, Table S7). Zebrafish that would otherwise have become females develop as sex-reversed males when homozygous for mutations in *Fanconi anemia* genes (*fanc* genes) [32], [33], which are essential to repair DNA damage by homologous recombination [94]. *fancg* and other genes already known to function in sex development, should be explored along with *dmrt1* and *cyp21a2* in efforts to identify the genetic elements underlying the associations between in *sar5* and *sar16* and sex.

The sex-associated loci we identified, *sar3* and *sar4*, suggest important avenues for future functional investigations. The 1.5 LOD drop interval of *sar3* contains about 315 annotated protein-coding genes in Zv9. Within this interval lies *hsd17b1*, which encodes the enzyme hydroxysteroid (17-*â*) dehydrogenase-1 (Figure 6). Hsd17b1 converts low-activity estrone to high-activity 17*â*-estradiol in zebrafish and in mammalian ovarian granulosa cells [95], and thus, along with Cyp19a1 (aromatase) catalyzes the final steps in estradiol biosynthesis from theca cell-derived androgens [96], [97]. In zebrafish adult ovaries, *hsd17b1* is expressed in Stage I follicles and peaks at Stage II, in parallel with the expression of *cyp19a1a* aromatase [31], [98], [99]. The expression of *hsd17b1* has not yet been investigated during the times at which sex determination occurs in zebrafish. Our model for zebrafish sex determination is that a signal from meiotic oocytes developing in gonads of the juvenile hermaphrodite stage of all individuals causes the soma to maintain the production of estrogen, which preserves ovary development and female differentiation; this suggests the hypothesis that differential regulation of *hsd17b1* is important in zebrafish sex determination. In humans, mutations altering the Hsd17b3 enzyme, which performs a similar reaction in the production of testosterone and is expressed in the mammalian testis and in vitellogenic ovarian follicles in zebrafish, cause reduced masculinization in childhood but virilization in adulthood; women (lacking testes) are asymptomatic [100].

The association between sex and the end of chr-4 (*sar4*) was observed in both Family A and Family B. Clearly, the identification of the molecular genetic basis of *sar4* is an important research goal, as is the discovery of the way in which it works to influence gonadal development. Unfortunately, an examination of the annotated genes in *sar4* does not provide an obvious strong candidate for a major sex determination gene (Figure 6 and Table S7). Within the 1.5 LOD drop region around *sar4* lie four 5S rRNA genes, three other ncRNA genes, and 56 annotated protein-coding elements. Most of the annotated protein-coding genes are members of large families, especially zinc finger domain families. Only nine protein-coding genes in *sar4* are annotated in Ensembl as orthologs of specific human genes: annotated as *PSMB10, PTCHD3 (2 of 3), gbp4, ttll1, pacsin2, arfgap3, IKBIP, CKAP4, terfa*, and *CCT2 (1 of 2)*. In mouse, *Ptchd3* is expressed specifically in meiotic spermatocytes [101], but the others, as far as is known, are not specifically expressed in gonads.

### Is chr-4 a Sex Chromosome?

Sex chromosomes represent a pair of originally homologous chromosomes that diverged during evolution due to the origin of a sex determining locus on one member of the pair [50], [102]–[105]. Sex chromosomes diverge by restricting recombination, initially in the region containing the sex determining locus (*e.g.*, in medaka [73]), and then gradually spreading along the chromosome; eventually, the chromosome accumulates transposable elements and pseudogenes [74]–[76].

We identified a sex-associated locus on zebrafish chr-4, a chromosome that shares characteristics with sex chromosomes in other species. A key feature of chr-4 that is shared with known sex determining chromosomal regions is suppressed recombination [73]–[76]. We found that recombination near *sar4* is substantially reduced in males relative to females (Figure 4). Because recombination between sex chromosomes is usually repressed in the heterogametic sex, this result might indicate that males are the heterogametic sex in zebrafish. Interestingly however, it has been proposed that sex determination in zebrafish is controlled by female-dominant genetic factors, as if zebrafish had a ZW/ZZ not an XX/XY sex-determining system [19]. The polygenic nature of sex determination in zebrafish (herein and [23]), might explain this apparent conflict. While chr-4 may be a sex chromosome, *sar4* is not the only sex-associated locus in zebrafish. Genes at *sar3*, *sar4*, *sar5*, and *sar16* may differ in whether they are female-dominant or male-dominant as is seen in sex-determining genes in cichlids [2]. Additional work must be done to determine how each of the 4 known zebrafish sex-associated regions influence sex and how they interact.

Chr-4 is also similar to known sex chromosomes in that it is late replicating, highly heterochromatic, and repeat rich (Figure 5, Table 2). Chr-4q contains a heterochromatic region that is approximately 34 Mb long. This region is enriched for genetic elements, including 5S RNA motifs, miRNA repeats, a number of repetitive gene families, snRNA genes, tRNA genes, the transposable element Kolobok-1, and pseudogenes. Sex chromosomes often accumulate transposable elements [74]–[76], [106]. Interestingly, chr-4 is not enriched for transposable elements *per se*, (see Figure S2), but contains multiple copies of Kolobok 1, a member of the autonomous Kolobok transposon family [81]. Chr-4 is also unique in having more than twice the average percentage of its chromosome length associated with copy number variant elements [84].

One interesting repetitive element on chr-4 is the cluster of 55 members of the *mir-430* gene family at about 28.0 Mb. Similar *mir-430* clusters are found in other teleost genomes, *e.g.* on medaka chr-4 and stickleback chromosome-IV. *Mir-430* helps to limit Nanos1 and TDRD7 proteins to primordial germ cells (PGCs) in zebrafish [107] and similar results have been found for medaka [108]. *Mir-430* also regulates the production of Sdf1a, a ligand secreted by somatic tissues that specifies the path along which primary germ cells (PGCs) migrate to the site of the future gonad during gastrulation [109]. Inhibiting the regulation of *sdf1a* leads to the mis-localization of germ cells, with fewer germ cells migrating to the gonad. In several species of fish, germ cell number appears to be an important variable distinguishing the development of an ovary (more PGCs) from a testis (fewer PGCs), and the strength of the PGC homing process could control the number of germ cells in the gonad [110]–[112].

According to our genomic analysis, chr-4q appears to be a mature sex chromosome, despite the fact that *sar4* is not as strong of a sex determinant as is *Sry* in mammals or *dmrt1by* in medaka. Thus, we speculate that chr-4q is an aging sex chromosome and that zebrafish is evolving new loci, like *sar3, sar5,* or *sar16* that may be variously emerging as new, young sex determinants. It is urgent to examine species closely related to the zebrafish *Danio rerio* to test this hypothesis.

### Conclusions

Our results reveal that zebrafish has a polygenic sex determining system with an important determinant occupying a short 2 Mb region at the tip of chr-4q, the only arm in the zebrafish genome that shares many features of sex chromosomes. Although several loci have been identified that affect zebrafish sex determination, as yet, no specific genes have been shown to be responsible for the sex-associated effects at any of the loci. Identification of these genes represents an important research agenda.

## Supporting Information

Figure S1 Genetic maps for Family A and Family B. Each vertical column represents one of the 25 zebrafish chromosomes. Each horizontal bar represents the location of a RAD-tag marker on the genetic map. Red rectangles approximate the location of sex-associated regions.
(TIF)

Figure S2 The distribution of repetitive elements and Kolobok transposons in the zebrafish genome and on chr-4 specifically. A. Distribution of all repeats and Kolobok elements across the entire genome. B. Distribution of all repeats and Kolobok elements across chr-4. C. Ideogram showing the location of the miRNA-430 gene cluster, the heterochromatic region of chr-4, and *sar4*. D. Number of Kolobok elements in the genome (# genome), in the heterochromatic region of chr-4 (# het region Dre4), and % of each Kolobok element in the heterochromatic region of chr-4 (% het region).
(TIF)

Table S1 Characteristics of polymorphic RAD-tag markers used for genetic mapping in Family A.
(XLSX)

Table S2 Characteristics of polymorphic RAD-tag markers used for genetic mapping in Family B.
(XLSX)

Table S3 Counts of female and male F2 offspring in each haplotype category for *sar3* (Table S4, Family B), and *sar4* (Tables S3 and S4, Family A and Family B). These data were used to calculate the percent male values presented in Figure 2 A, B.
(DOC)

Table S4 Counts of female and male F2 offspring in each haplotype category for *sar3* (Table S4, Family B), and *sar4* (Tables S3 and S4, Family A and Family B). These data were used to calculate the percent male values presented in Figure 2 A, B.
(DOC)

Table S5 Details for SNPs found at significantly different frequencies in females and males including chromosome, position in the Zv9 genome assembly, G-test statistic and associated p-value, observed alleles (alleles 1 and 2), the numbers of females and males of each genotype, and the total numbers of individuals scored.
(XLSX)

Table S6 Details for SNPs found at significantly different frequencies in females and males including chromosome, position in the Zv9 genome assembly, G-test statistic and associated p-value, observed alleles (alleles 1 and 2), the numbers of females and males of each genotype, and the total numbers of individuals scored.
(XLSX)

Table S7 Genomic locations of candidate genes for sex determination in the zebrafish genome (genome assembly Zv9, Ensembl version 65).
(XLSX)

Text S1 A *Fixer.py* custom script that recodes genotypes from JoinMap family type CP for analysis as a phase-known 4-way cross in R/qtl.
(DOCX)

Text S2 Partial sequence of *dmrt1* from *Danio rerio*. Heterozygous positions noted using IUPAC ambiguity code. Orange boxes indicate sequence associated with ss184964113 and ss184964140 in the dbSNP database at NCBI (http://www.ncbi.nlm.nih.gov/projects/SNP/), the SNPs identified as potentially functional by Bradley et al. (2011).
(DOCX)
