# Supplementary figures and images for: Multiple Sex-Associated Regions and a Putative Sex Chromosome in Zebrafish Revealed by RAD Mapping and Population Genomics
Source: PLoS One. 2012 Jul 9;7(7):e40701. doi: 10.1371/journal.pone.0040701 (PMC3392230; doi:10.1371/journal.pone.0040701)

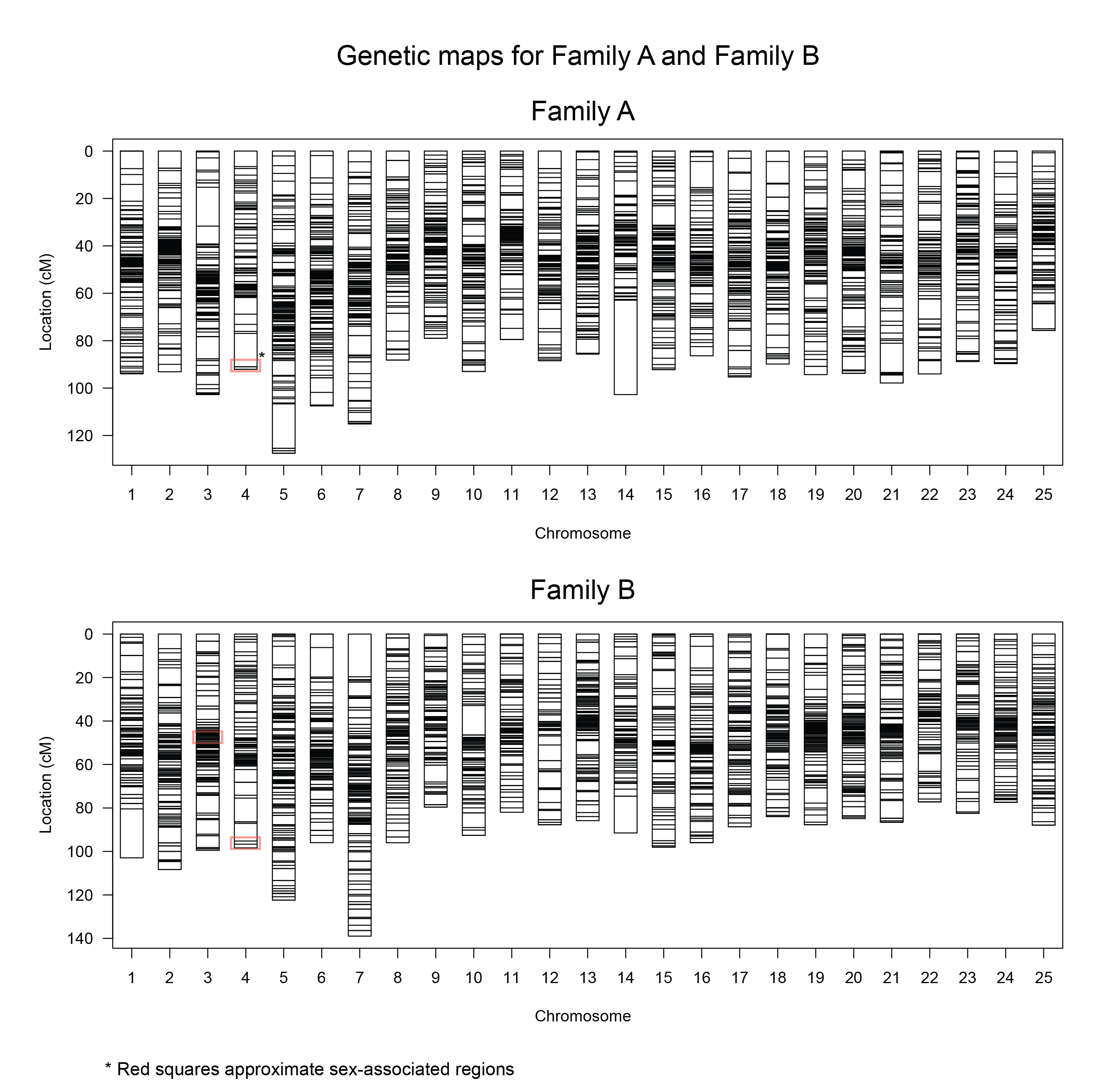

Supplement: Figure S1 — Genetic maps for Family A and Family B. Each vertical column represents one of the 25 zebrafish chromosomes. Each horizontal bar represents the location of a RAD-tag marker on the genetic map. Red rectangles approximate the location of sex-associated regions. (TIF) [file pone.0040701.s001.tif]

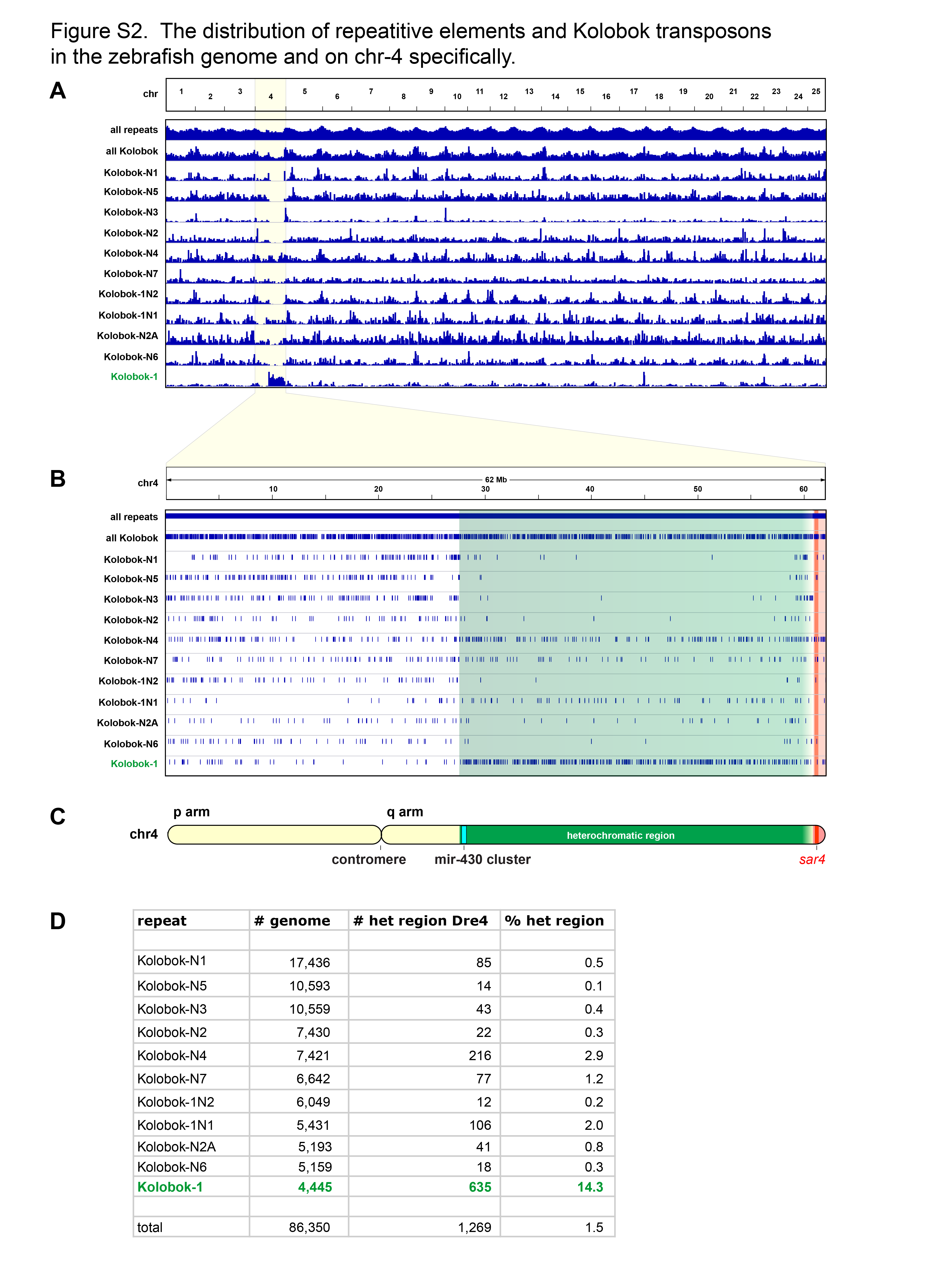

Supplement: Figure S2 — The distribution of repetitive elements and Kolobok transposons in the zebrafish genome and on chr-4 specifically. A. Distribution of all repeats and Kolobok elements across the entire genome. B. Distribution of all repeats and Kolobok elements across chr-4. C. Ideogram showing the location of the miRNA-430 gene cluster, the heterochromatic region of chr-4, and sar4. D. Number of Kolobok elements in the genome (# genome), in the heterochromatic region of chr-4 (# het region Dre4), and % of each Kolobok element in the heterochromatic region of chr-4 (% het region). (TIF) [file pone.0040701.s002.tif]
