# Supplementary material for: Multiple Sex-Associated Regions and a Putative Sex Chromosome in Zebrafish Revealed by RAD Mapping and Population Genomics
Source: PLoS One. 2012 Jul 9;7(7):e40701. doi: 10.1371/journal.pone.0040701 (PMC3392230; doi:10.1371/journal.pone.0040701)
Supplement: Table S3 — Counts of female and male F2 offspring in each haplotype category for sar3 (Table S4, Family B), and sar4 (Tables S3 and S4, Family A and Family B). These data were used to calculate the percent male values presented in Figure 2 A, B. (DOC) [file pone.0040701.s005.doc]

Tables S3. Counts of female and male *F_2_* offspring in each haplotype category for *sar3* (Table S4, Family B), and *sar4* (Tables S3 and S4, Family A and Family B). These data were used to calculate the percent male values presented in Figure 2 A, B.

| Table S3. Counts of female and male *F_2_* offspring in each haplotype category for Family A | | |
| --- | --- | --- |
| Family A |  |  |
| Chr-4 haplotype, marker ID29464 near Zv9 physical position 4:61,176,889bp | female | male |
| C/C | 0 | 70 |
| C/G | 63 | 12 |
