# Supplementary material for: Multiple Sex-Associated Regions and a Putative Sex Chromosome in Zebrafish Revealed by RAD Mapping and Population Genomics
Source: PLoS One. 2012 Jul 9;7(7):e40701. doi: 10.1371/journal.pone.0040701 (PMC3392230; doi:10.1371/journal.pone.0040701)
Supplement: Table S4 — Counts of female and male F2 offspring in each haplotype category for sar3 (Table S4, Family B), and sar4 (Tables S3 and S4, Family A and Family B). These data were used to calculate the percent male values presented in Figure 2 A, B. (DOC) [file pone.0040701.s006.doc]

| Table S4. Counts of female and male *F_2_* offspring in each haplotype category for Family B. | | | | | | | | |
| --- | --- | --- | --- | --- | --- | --- | --- | --- |
| Family B |  | | | | | | | |
|  | Chr-4 haplotype, marker ID32525 near physical position 4:61,422,807bp | | | | | | | |
| Chr-3  Haplotype, marker ID29552, position 3:20,676,406bp | ACA/CCG | | ACA/CTA | | CCG/ CCG | | CCG/CTA | |
|  | female | male | female | male | female | male | female | male |
| AG/AG | 0 | 7 | 0 | 5 | 0 | 5 | 0 | 3 |
| AG/CC | 0 | 6 | 8 | 6 | 10 | 0 | 8 | 0 |
| CC/CC | 0 | 3 | 2 | 3 | 1 | 1 | 4 | 1 |

Table S4. Counts of female and male *F_2_* offspring in each haplotype category for *sar3* (Table S4, Family B), and *sar4* (Tables S3 and S4, Family A and Family B). These data were used to calculate the percent male values presented in Figure 2 A, B.
