# Supplementary material for: Multiple Sex-Associated Regions and a Putative Sex Chromosome in Zebrafish Revealed by RAD Mapping and Population Genomics
Source: PLoS One. 2012 Jul 9;7(7):e40701. doi: 10.1371/journal.pone.0040701 (PMC3392230; doi:10.1371/journal.pone.0040701)
Supplement: Text S1 — A Fixer.py custom script that recodes genotypes from JoinMap family type CP for analysis as a phase-known 4-way cross in R/qtl. (DOCX) [file pone.0040701.s010.docx]

Text S1. Fixer.py custom script that recodes genotypes from JoinMap family type CP for analysis as a phase known 4-way cross in R/qtl.

#Written by Peter Batzel

#Nov, 2011

import sys

if (len(sys.argv) !=3):

sys.exit("You did not pass the correct arguments: fixer.py infile outfile")

else:

in_handle = sys.argv[1]

out_handle = sys.argv[2]

#Always write the first two lines

inputFile = open(in_handle, 'rU')

outputFile = open(out_handle, 'w')

line = inputFile.readline()

outputFile.write(line)

line = inputFile.readline()

outputFile.write(line)

outString = ""

#Loop through to the end of the inputFile

while True:

line = inputFile.readline().strip()

if(line == ""):

break

line = line.split('(')[0]+ line.split(')')[1]

lineSplit = line.split(",")

x = 5

#Loop through the elements from this line

while x < len(lineSplit):

if ((lineSplit[3] == "{0-}")):

if(lineSplit[x] == "ll"):

lineSplit[x] = 5

elif(lineSplit[x] == "lm"):

lineSplit[x] = 6

elif(lineSplit[3] == "{1-}"):

if(lineSplit[x] == 'lm'):

lineSplit[x] = 5

elif(lineSplit[x] =='ll'):

lineSplit[x] = 6

elif(lineSplit[3] == "{-0}"):

if(lineSplit[x] == 'nn'):

lineSplit[x] = 7

elif(lineSplit[x] == 'np'):

lineSplit[x] = 8

elif(lineSplit[3] == "{-1}"):

if(lineSplit[x] == 'np'):

lineSplit[x] = 7

elif(lineSplit[x] == 'nn'):

lineSplit[x] = 8

elif(lineSplit[3] == "{00}"):

if(lineSplit[x] == 'ee'):

lineSplit[x] = 1

elif(lineSplit[x] == 'ef'):

lineSplit[x] = 2

elif(lineSplit[x] == 'eg'):

lineSplit[x] = 3

elif(lineSplit[x] == 'fg'):

lineSplit[x] =4

elif(lineSplit[x] == 'hh'):

lineSplit[x] = 1

elif(lineSplit[x] == 'hk'):

lineSplit[x] = 10

elif(lineSplit[3] == "{10}"):

if(lineSplit[x] == 'ef'):

lineSplit[x] = 1

elif(lineSplit[x] == 'ee'):

lineSplit[x] = 2

elif(lineSplit[x] == 'fg'):

lineSplit[x] = 3

elif(lineSplit[x] == 'eg'):

lineSplit[x] = 4

elif(lineSplit[x] == 'hk'):

lineSplit[x] = 9

elif(lineSplit[x] == 'hh'):

lineSplit[x] = 2

elif(lineSplit[3] == "{01}"):

if(lineSplit[x] == 'eg'):

lineSplit[x] = 1

elif(lineSplit[x] == 'fg'):

lineSplit[x] = 2

elif(lineSplit[x] == 'ee'):

lineSplit[x] = 3

elif(lineSplit[x] == 'ef'):

lineSplit[x] = 4

elif(lineSplit[x] == 'hk'):

lineSplit[x] = 9

elif(lineSplit[x] == 'kk'):

lineSplit[x] = 2

elif(lineSplit[3] == "{11}"):

if(lineSplit[x] == 'fg'):

lineSplit[x] = 1

elif(lineSplit[x] == 'eg'):

lineSplit[x] = 2

elif(lineSplit[x] == 'ef'):

lineSplit[x] = 3

elif(lineSplit[x] == 'ee'):

lineSplit[x] = 4

else: #According to our logic, this shouldn't be able to happen. If it does, some additional logic will need to be added.

sys.exit("Found hkxhk with phase {11}")

else:

if(lineSplit[x] == 'ac'):

lineSplit[x] = 1

elif(lineSplit[x] == 'bc'):

lineSplit[x] = 2

elif(lineSplit[x] == 'ad'):

lineSplit[x] = 3

elif(lineSplit[x] == 'bd'):

lineSplit[x] = 4

x += 1

outString = (str(lineSplit))

outputFile.write(outString[1:-1]+'\n')

print "Done"

inputFile.close()

outputFile.close()
