# Supplementary material for: Multiple Sex-Associated Regions and a Putative Sex Chromosome in Zebrafish Revealed by RAD Mapping and Population Genomics
Source: PLoS One. 2012 Jul 9;7(7):e40701. doi: 10.1371/journal.pone.0040701 (PMC3392230; doi:10.1371/journal.pone.0040701)
Supplement: Text S2 — Partial sequence of dmrt1 from Danio rerio. Heterozygous positions noted using IUPAC ambiguity code. Orange boxes indicate sequence associated with ss184964113 and ss184964140 in the dbSNP database at NCBI (http://www.ncbi.nlm.nih.gov/projects/SNP/), the SNPs identified as potentially functional by Bradley et al. (2011). (DOCX) [file pone.0040701.s011.docx]

Text S2. Partial sequence of *dmrt1* from *Danio rerio*. Heterozygous positions noted using IUPAC ambiguity code. Orange boxes indicate sequence associated with ss184964113 and ss184964140 in the dbSNP database at NCBI (<http://www.ncbi.nlm.nih.gov/projects/SNP/>)

1 10 20 30 40 50 60

| | | | | | |

AB-01 female TAAAAGAAGTCTCCAATAATAAACACCAAATAGTTCATGTTTCATGATCCATCCCATTTC

AB-02 female TAAAAGAAGTCTCCAATAATAAACACCAAATAGTTCATGTTTCATGATCCATCCCATTTC

AB-03 female TAAAAGAAGTCTCCAATAATAAACACCAAATAGTTCATGTTTCATGATCCATCCCATTTC

AB-04 female TAAAAGAAGTCTCCAATAATAAACACCAAATAGTTCATGTTTCATGATCCATCCCATTTC

AB-05 female TAAAAGAAGTCTCCAATAATAAACACCAAATAGTTCATGTTTCATGATCCATCCCATTTC

AB-06 female TAAAAGAAGTCTCCAATAATAAACACCAAATAGTTCATGTTTCATGATCCATCCCATTTC

AB-07 female TAAAAGAAGTCTCCAATAATAAACACCAAATAGTTCATGTTTCATGATCCATCCCATTTC

AB-08 female TAAAAGAAGTCTCCAATAATAAACACCAAATAGTTCATGTTTCATGATCCATCCCATTTC

AB-09 female TAAAAGAAGTCTCCAATAATAAACACCAAATAGTTCATGTTTCATGATCCATCCCATTTC

AB-10 female TAAAAGAAGTCTCCAATAATAAACACCAAATAGTTCATGTTTCATGATCCATCCCATTTC

AB-11 female TAAAAGAAGTCTCCAATAATAAACACCAAATAGTTCATGTTTCATGATCCATCCCATTTC

AB-12 female TAAAAGAAGTCTCCAATAATAAACACCAAATAGTTCATGTTTCATGATCCATCCCATTTC

AB-13 male TAAAAGAAGTCTCCAATAATAAACACCAAATAGTTCATGTTTCATGATCCATCCCATTTC

AB-14 male TAAAAGAAGTCTCCAATAATAAACACCAAATAGTTCATGTTTCATGATCCATCCCATTTC

AB-15 male TAAAAGAAGTCTCCAATAATAAACACCAAATAGTTCATGTTTCATGATCCATCCCATTTC

AB-16 male TAAAAGAAGTCTCCAATAATAAACACCAAATAGTTCATGTTTCATGATCCATCCCATTTC

AB-17 male TAAAAGAAGTCTCCAATAATNAACACCAAATAGTTCATGTTTCATGATCCATCCCATTTC

AB-18 male TAAAAGAAGTCTCCAATAATAAACACCAAATAGTTCATGTTTCATGATCCATCCCATTTC

AB-19 male TAAAAGAAGTCTCCAATAATAAACACCAAATAGTTCATGTTTCATGATCCATCCCATTTC

AB-20 male TAAAAGAAGTCTCCAATAATAAACACCAAATAGTTCATGTTTCATGATCCATCCCATTTC

AB-21 male TAAAAGAAGTCTCCAATAATAAACACCAAATAGTTCATGTTTCATGATCCATCCCATTTC

AB-22 male TAAAAGAAGTCTCCAATAATAAACACCAAATAGTTCATGTTTCATGATCCATCCCATTTC

AB-23 male TAAAAGAAGTCTCCAATAATAAACACCAAATAGTTCATGTTTCATGATCCATCCCATTTC

AB-24 male TAAAAGAAGTCTCCAATAATAAACACCAAATAGTTCATGTTTCATGATCCATCCCATTTC

AB-01 female AGCTTTCTCCGATGGAGCTCAAGACTCTGTGTCCATCAGCTCGATGATCAACGCTGAGAA

AB-02 female AGCTTTCTCCGATGGAGCTCAAGACTCTGTGTCCATCAGCTCGATGATCAACGCTGAGAA

AB-03 female AGCTTTCTCCGATGGAGCTCAAGACTCTGTGTCCATCAGCTCGATGATCAACGCTGAGAA

AB-04 female AGCTTTCTCCGATGGAGCTCAAGACTCTGTGTCCATCAGCTCGATGATCAACGCTGAGAA

AB-05 female AGCTTTCTCCGATGGAGCTCAAGACTCTGTGTCCATCAGCTCGATGATCAACGCTGAGAA

AB-06 female AGCTTTCTCCGATGGAGCTCAAGACTCTGTGTCCATCAGCTCGATGATCAACGCTGAGAA

AB-07 female AGCTTTCTCCGATGGAGCTCAAGACTCTGTGTCCATCAGCTCGATGATCAACGCTGAGAA

AB-08 female AGCTTTCTCCGATGGAGCTCAAGACTCTGTGTCCATCAGCTCGATGATCAACGCTGAGAA

AB-09 female AGCTTTCTCCGATGGAGCTCAAGACTCTGTGTCCATCAGCTCGATGATCAACGCTGAGAA

AB-10 female AGCTTTCTCCGATGGAGCTCAAGACTCTGTGTCCATCAGCTCGATGATCAACGCTGAGAA

AB-11 female AGCTTTCTCCGATGGAGCTCAAGACTCTGTGTCCATCAGCTCGATGATCAACGCTGAGAA

AB-12 female AGCTTTCTCCGATGGAGCTCAAGACTCTGTGTCCATCAGCTCGATGATCAACGCTGAGAA

AB-13 male AGCTTTCTCCGATGGAGCTCAAGACTCTGTGTCCATCAGCTCGATGATCAACGCTGAGAA

AB-14 male AGCTTTCTCCGATGGAGCTCAAGACTCTGTGTCCATCAGCTCGATGATCAACGCTGAGAA

AB-15 male AGCTTTCTCCGATGGAGCTCAAGACTCTGTGTCCATCAGCTCGATGATCAACGCTGAGAA

AB-16 male AGCTTTCTCCGATGGAGCTCAAGACTCTGTGTCCATCAGCTCGATGATCAACGCTGAGAA

AB-17 male AGCTTTCTCCGATGGAGCTCAAGACTCTGTGTCCATCAGCTCGATGATCAACGCTGAGAA

AB-18 male AGCTTTCTCCGATGGAGCTCAAGACTCTGTGTCCATCAGCTCGATGATCAACGCTGAGAA

AB-19 male AGCTTTCTCCGATGGAGCTCAAGACTCTGTGTCCATCAGCTCGATGATCAACGCTGAGAA

AB-20 male AGCTTTCTCCGATGGAGCTCAAGACTCTGTGTCCATCAGCTCGATGATCAACGCTGAGAA

AB-21 male AGCTTTCTCCGATGGAGCTCAAGACTCTGTGTCCATCAGCTCGATGATCAACGCTGAGAA

AB-22 male AGCTTTCTCCGATGGAGCTCAAGACTCTGTGTCCATCAGCTCGATGATCAACGCTGAGAA

AB-23 male AGCTTTCTCCGATGGAGCTCAAGACTCTGTGTCCATCAGCTCGATGATCAACGCTGAGAA

AB-24 male AGCTTTCTCCGATGGAGCTCAAGACTCTGTGTCCATCAGCTCGATGATCAACGCTGAGAA

1 10 20 30 40 50 60

| | | | | | |

AB-01 female CAAGCTGGAGTGTGAAAGCAGTTCAGAGTCTGGAAGCTTCTCAGTCGACTCTATCATAGA

AB-02 female CAAGCTGGAGTGTGAAAGCAGTTCAGAGTCTGGAAGCTTCTCAGTCGACTCYATCATAGA

AB-03 female CAAGCTGGAGTGTGAAAGCAGTTCAGAGTCTGGAAGCTTCTCAGTCGACTCYATCATAGA

AB-04 female CAAGCTGGAGTGTGAAAGCAGTTCAGAGTCTGGAAGCTTCTCAGTCGACTCYATCATAGA

AB-05 female CAAGCTGGAGTGTGAAAGCAGTTCAGAGTCTGGAAGCTTCTCAGTCGACTCTATCATAGA

AB-06 female CAAGCTGGAGTGTGAAAGCAGTTCAGAGTCTGGAAGCTTCTCAGTCGACTCYATCATAGA

AB-07 female CAAGCTGGAGTGTGAAAGCAGTTCAGAGTCTGGAAGCTTCTCAGTCGACTCYATCATAGA

AB-08 female CAAGCTGGAGTGTGAAAGCAGTTCAGAGTCTGGAAGCTTCTCAGTCGACTCYATCATAGA

AB-09 female CAAGCTGGAGTGTGAAAGCAGTTCAGAGTCTGGAAGCTTCTCAGTCGACTCYATCATAGA

AB-10 female CAAGCTGGAGTGTGAAAGCAGTTCAGAGTCTGGAAGCTTCTCAGTCGACTCYATCATAGA

AB-11 female CAAGCTGGAGTGTGAAAGCAGTTCAGAGTCTGGAAGCTTCTCAGTCGACTCYATCATAGA

AB-12 female CAAGCTGGAGTGTGAAAGCAGTTCAGAGTCTGGAAGCTTCTCAGTCGACTCTATCATAGA

AB-13 male CAAGCTGGAGTGTGAAAGCAGTTCAGAGTCTGGAAGCTTCTCAGTCGACTCTATCATAGA

AB-14 male CAAGCTGGAGTGTGAAAGCAGTTCAGAGTCTGGAAGCTTCTCAGTCGACTCYATCATAGA

AB-15 male CAAGCTGGAGTGTGAAAGCAGTTCAGAGTCTGGAAGCTTCTCAGTCGACTCTATCATAGA

AB-16 male CAAGCTGGAGTGTGAAAGCAGTTCAGAGTCTGGAAGCTTCTCAGTCGACTCTATCATAGA

AB-17 male CAAGCTGGAGTGTGAAAGCAGTTCAGAGTCTGGAAGCTTCTCAGTCGACTCYATCATAGA

AB-18 male CAAGCTGGAGTGTGAAAGCAGTTCAGAGTCTGGAAGCTTCTCAGTCGACTCYATCATAGA

AB-19 male CAAGCTGGAGTGTGAAAGCAGTTCAGAGTCTGGAAGCTTCTCAGTCGACTCYATCATAGA

AB-20 male CAAGCTGGAGTGTGAAAGCAGTTCAGAGTCTGGAAGCTTCTCAGTCGACTCTATCATAGA

AB-21 male CAAGCTGGAGTGTGAAAGCAGTTCAGAGTCTGGAAGCTTCTCAGTCGACTCYATCATAGA

AB-22 male CAAGCTGGAGTGTGAAAGCAGTTCAGAGTCTGGAAGCTTCTCAGTCGACTCTATCATAGA

AB-23 male CAAGCTGGAGTGTGAAAGCAGTTCAGAGTCTGGAAGCTTCTCAGTCGACTCTATCATAGA

AB-24 male CAAGCTGGAGTGTGAAAGCAGTTCAGAGTCTGGAAGCTTCTCAGTCGACTCTATCATAGA

ss184964113

AB-01 female GGGGGCCACCAAATGAAAGACCATCTCCAGCTGCCAGTTACAACAACTTGAATTTTTTGT

AB-02 female GGGGGCCACCAAATGAAAGACCATCTCCAGCTGCCAGTTACAACAACTTGAAYTTTTTGT

AB-03 female GGGGGCCACCAAATGAAAGACCATCTCCAGCTGCCAGTTACAACAACTTGAAYTTTTTGT

AB-04 female GGGGGCCACCAAATGAAAGACCATCTCCAGCTGCCAGTTACAACAACTTGAAYTTTTTGT

AB-05 female GGGGGCCACCAAATGAAAGACCATCTCCAGCTGCCAGTTACAACAACTTGAATTTTTTGT

AB-06 female GGGGGCCACCAAATGAAAGACCATCTCCAGCTGCCAGTTACAACAACTTGAAYTTTTTGT

AB-07 female GGGGGCCACCAAATGAAAGACCATCTCCAGCTGCCAGTTACAACAACTTGAAYTTTTTGT

AB-08 female GGGGGCCACCAAATGAAAGACCATCTCCAGCTGCCAGTTACAACAACTTGAAYTTTTTGT

AB-09 female GGGGGCCACCAAATGAAAGACCATCTCCAGCTGCCAGTTACAACAACTTGAAYTTTTTGT

AB-10 female GGGGGCCACCAAATGAAAGACCATCTCCAGCTGCCAGTTACAACAACTTGAAYTTTTTGT

AB-11 female GGGGGCCACCAAATGAAAGACCATCTCCAGCTGCCAGTTACAACAACTTGAAYTTTTTGT

AB-12 female GGGGGCCACCAAATGAAAGACCATCTCCAGCTGCCAGTTACAACAACTTGAATTTTTTGT

AB-13 male GGGGGCCACCAAATGAAAGACCATCTCCAGCTGCCAGTTACAACAACTTGAATTTTTTGT

AB-14 male GGGGGCCACCAAATGAAAGACCATCTCCAGCTGCCAGTTACAACAACTTGAAYTTTTTGT

AB-15 male GGGGGCCACCAAATGAAAGACCATCTCCAGCTGCCAGTTACAACAACTTGAATTTTTTGT

AB-16 male GGGGGCCACCAAATGAAAGACCATCTCCAGCTGCCAGTTACAACAACTTGAATTTTTTGT

AB-17 male GGGGGCCACCAAATGAAAGACCATCTCCAGCTGCCAGTTACAACAACTTGAAYTTTTTGT

AB-18 male GGGGGCCACCAAATGAAAGACCATCTCCAGCTGCCAGTTACAACAACTTGAAYTTTTTGT

AB-19 male GGGGGCCACCAAATGAAAGACCATCTCCAGCTGCCAGTTACAACAACTTGAAYTTTTTGT

AB-20 male GGGGGCCACCAAATGAAAGACCATCTCCAGCTGCCAGTTACAACAACTTGAATTTTTTGT

AB-21 male GGGGGCCACCAAATGAAAGACCATCTCCAGCTGCCAGTTACAACAACTTGAAYTTTTTGT

AB-22 male GGGGGCCACCAAATGAAAGACCATCTCCAGCTGCCAGTTACAACAACTTGAATTTTTTGT

AB-23 male GGGGGCCACCAAATGAAAGACCATCTCCAGCTGCCAGTTACAACAACTTGAATTTTTTGT

AB-24 male GGGGGCCACCAAATGAAAGACCATCTCCAGCTGCCAGTTACAACAACTTGAATTTTTTGT

1 10 20 30 40 50 60

| | | | | | |

AB-01 female TAAATGAGAAGTTTCTGTTATTTTCAGCCAAATGTGTAATGATTCTGTAATCAACATGTT

AB-02 female TAAATGAGAAGTTWCTGTTATTTTCAGCCAAATGTGTAATGATTCTGTAAYCAACATGTT

AB-03 female TAAATGAGAAGTTWCTGTTATTTTCAGCCAAATGTGTAATGATTCTGTAAYCAACATGTT

AB-04 female TAAATGAGAAGTTWCTGTTATTTTCAGCCAAATGTGTAATGATTCTGTAAYCAACATGTT

AB-05 female TAAATGAGAAGTTTCTGTTATTTTCAGCCAAATGTGTAATGATTCTGTAATCAACATGTT

AB-06 female TAAATGAGAAGTTWCTGTTATTTTCAGCCAAATGTGTAATGATTCTGTAAYCAACATGTT

AB-07 female TAAATGAGAAGTTWCTGTTATTTTCAGCCAAATGTGTAATGATTCTGTAAYCAACATGTT

AB-08 female TAAATGAGAAGTTWCTGTTATTTTCAGCCAAATGTGTAATGATTCTGTAAYCAACATGTT

AB-09 female TAAATGAGAAGTTWCTGTTATTTTCAGCCAAATGTGTAATGATTCTGTAAYCAACATGTT

AB-10 female TAAATGAGAAGTTWCTGTTATTTTCAGCCAAATGTGTAATGATTCTGTAAYCAACATGTT

AB-11 female TAAATGAGAAGTTWCTGTTATTTTCAGCCAAATGTGTAATGATTCTGTAAYCAACATGTT

AB-12 female TAAATGAGAAGTTTCTGTTATTTTCAGCCAAATGTGTAATGATTCTGTAATCAACATGTT

AB-13 male TAAATGAGAAGTTTCTGTTATTTTCAGCCAAATGTGTAATGATTCTGTAATCAACATGTT

AB-14 male TAAATGAGAAGTTWCTGTTATTTTCAGCCAAATGTGTAATGATTCTGTAAYCAACATGTT

AB-15 male TAAATGAGAAGTTTCTGTTATTTTCAGCCAAATGTGTAATGATTCTGTAATCAACATGTT

AB-16 male TAAATGAGAAGTTTCTGTTATTTTCAGCCAAATGTGTAATGATTCTGTAATCAACATGTT

AB-17 male TAAATGAGAAGTTWCTGTTATTTTCAGCCAAATGTGTAATGATTCTGTAAYCAACATGTT

AB-18 male TAAATGAGAAGTTWCTGTTATTTTCAGCCAAATGTGTAATGATTCTGTAAYCAACATGTT

AB-19 male TAAATGAGAAGTTWCTGTTATTTTCAGCCAAATGTGTAATGATTCTGTAAYCAACATGTT

AB-20 male TAAATGAGAAGTTTCTGTTATTTTCAGCCAAATGTGTAATGATTCTGTAATCAACATGTT

AB-21 male TAAATGAGAAGTTWCTGTTATTTTCAGCCAAATGTGTAATGATTCTGTAAYCAACATGTT

AB-22 male TAAATGAGAAGTTTCTGTTATTTTCAGCCAAATGTGTAATGATTCTGTAATCAACATGTT

AB-23 male TAAATGAGAAGTTTCTGTTATTTTCAGCCAAATGTGTAATGATTCTGTAATCAACATGTT

AB-24 male TAAATGAGAAGTTTCTGTTATTTTCAGCCAAATGTGTAATGATTCTGTAATCAACATGTT

AB-01 female TCCAATAATACTGATCAGGTTGATCACAGTGCTGTTTTTATTCTCTTTTTAGGGGTCGTT

AB-02 female TCCAATAATACTGATCAGGTTGATCACAGTGCTGTTTTTWTTCTCTTTTTAGGGGTCGTT

AB-03 female TCCAATAATACTGATCAGGTTGATCACAGTGCTGTTTTTWTTCTCTTTTTAGGGGTCGTT

AB-04 female TCCAATAATACTGATCAGGTTGATCACAGTGCTGTTTTTWTTCTCTTTTTAGGGGTCGTT

AB-05 female TCCAATAATACTGATCAGGTTGATCACAGTGCTGTTTTTATTCTCTTTTTAGGGGTCGTT

AB-06 female TCCAATAATACTGATCAGGTTGATCACAGTGCTGTTTTTWTTCTCTTTTTAGGGGTCGTT

AB-07 female TCCAATAATACTGATCAGGTTGATCACAGTGCTGTTTTTWTTCTCTTTTTAGGGGTCGTT

AB-08 female TCCAATAATACTGATCAGGTTGATCACAGTGCTGTTTTTWTTCTCTTTTTAGGGGTCGTT

AB-09 female TCCAATAATACTGATCAGGTTGATCACAGTGCTGTTTTTWTTCTCTTTTTAGGGGTCGTT

AB-10 female TCCAATAATACTGATCAGGTTGATCACAGTGCTGTTTTTWTTCTCTTTTTAGGGGTCGTT

AB-11 female TCCAATAATACTGATCAGGTTGATCACAGTGCTGTTTTTWTTCTCTTTTTAGGGGTCGTT

AB-12 female TCCAATAATACTGATCAGGTTGATCACAGTGCTGTTTTTATTCTCTTTTTAGGGGTCGTT

AB-13 male TCCAATAATACTGATCAGGTTGATCACAGTGCTGTTTTTATTCTCTTTTTAGGGGTCGTT

AB-14 male TCCAATAATACTGATCAGGTTGATCACAGTGCTGTTTTTWTTCTCTTTTTAGGGGTCGTT

AB-15 male TCCAATAATACTGATCAGGTTGATCACAGTGCTGTTTTTATTCTCTTTTTAGGGGTCGTT

AB-16 male TCCAATAATACTGATCAGGTTGATCACAGTGCTGTTTTTATTCTCTTTTTAGGGGTCGTT

AB-17 male TCCAATAATACTGATCAGGTTGATCACAGTGCTGTTTTTWTTCTCTTTTTAGGGGTCGTT

AB-18 male TCCAATAATACTGATCAGGTTGATCACAGTGCTGTTTTTWTTCTCTTTTTAGGGGTCGTT

AB-19 male TCCAATAATACTGATCAGGTTGATCACAGTGCTGTTTTTWTTCTCTTTTTAGGGGTCGTT

AB-20 male TCCAATAATACTGATCAGGTTGATCACAGTGCTGTTTTTATTCTCTTTTTAGGGGTCGTT

AB-21 male TCCAATAATACTGATCAGGTTGATCACAGTGCTGTTTTTWTTCTCTTTTTAGGGGTCGTT

AB-22 male TCCAATAATACTGATCAGGTTGATCACAGTGCTGTTTTTATTCTCTTTTTAGGGGTCGTT

AB-23 male TCCAATAATACTGATCAGGTTGATCACAGTGCTGTTTTTATTCTCTTTTTAGGGGTCGTT

AB-24 male TCCAATAATACTGATCAGGTTGATCACAGTGCTGTTTTTATTCTCTTTTTAGGGGTCGTT

1 10 20 30 40 50 60

| | | | | | |

AB-01 female CTATAAGGTTTTGACTGTTATTGTTAACTCGATTGTTGTCATCGATGACAAGAAATGCTG

AB-02 female CTATAAGGTTTTGACTKTTATTGTTAACTCGATTGTTGTCATCGATGACAAGAAATGCTG

AB-03 female CTATAAGGTTTTGACTKTTATTGTTAACTCGATTGTTGTCATCGATGACAAGAAATGCTG

AB-04 female CTATAAGGTTTTGACTKTTATTGTTAACTCGATTGTTGTCATCGATGACAAGAAATGCTG

AB-05 female CTATAAGGTTTTGACTGTTATTGTTAACTCGATTGTTGTCATCGATGACAAGAAATGCTG

AB-06 female CTATAAGGTTTTGACTKTTATTGTTAACTCGATTGTTGTCATCGATGACAAGAAATGCTG

AB-07 female CTATAAGGTTTTGACTKTTATTGTTAACTCGATTGTTGTCATCGATGACAAGAAATGCTG

AB-08 female CTATAAGGTTTTGACTKTTATTGTTAACTCGATTGTTGTCATCGATGACAAGAAATGCTG

AB-09 female CTATAAGGTTTTGACTKTTATTGTTAACTCGATTGTTGTCATCGATGACAAGAAATGCTG

AB-10 female CTATAAGGTTTTGACTKTTATTGTTAACTCGATTGTTGTCATCGATGACAAGAAATGCTG

AB-11 female CTATAAGGTTTTGACTKTTATTGTTAACTCGATTGTTGTCATCGATGACAAGAAATGCTG

AB-12 female CTATAAGGTTTTGACTGTTATTGTTAACTCGATTGTTGTCATCGATGACAAGAAATGCTG

AB-13 male CTATAAGGTTTTGACTGTTATTGTTAACTCGATTGTTGTCATCGATGACAAGAAATGCTG

AB-14 male CTATAAGGTTTTGACTKTTATTGTTAACTCGATTGTTGTCATCGATGACAAGAAATGCTG

AB-15 male CTATAAGGTTTTGACTGTTATTGTTAACTCGATTGTTGTCATCGATGACAAGAAATGCTG

AB-16 male CTATAAGGTTTTGACTGTTATTGTTAACTCGATTGTTGTCATCGATGACAAGAAATGCTG

AB-17 male CTATAAGGTTTTGACTKTTATTGTTAACTCGATTGTTGTCATCGATGACAAGAAATGCTG

AB-18 male CTATAAGGTTTTGACTKTTATTGTTAACTCGATTGTTGTCATCGATGACAAGAAATGCTG

AB-19 male CTATAAGGTTTTGACTKTTATTGTTAACTCGATTGTTGTCATCGATGACAAGAAATGCTG

AB-20 male CTATAAGGTTTTGACTGTTATTGTTAACTCGATTGTTGTCATCGATGACAAGAAATGCTG

AB-21 male CTATAAGGTTTTGACTKTTATTGTTAACTCGATTGTTGTCATCGATGACAAGAAATGCTG

AB-22 male CTATAAGGTTTTGACTGTTATTGTTAACTCGATTGTTGTCATCGATGACAAGAAATGCTG

AB-23 male CTATAAGGTTTTGACTGTTATTGTTAACTCGATTGTTGTCATCGATGACAAGAAATGCTG

AB-24 male CTATAAGGTTTTGACTGTTATTGTTAACTCGATTGTTGTCATCGATGACAAGAAATGCTG

ss184964140

AB-01 female TTTACATAGTTTACTAATATGTTTCACACAGTTTGATAGCTTAGCTTTGTTCACTTTACT

AB-02 female TTTACATAGTTTACTWATATGTTTCACACAGTTTGATAGCTTAGCTTTGTTCACTTTACT

AB-03 female TTTACATAGTTTACTWATATGTTTCACACAGTTTGATAGCTTAGCTTTGTTCACTTTACT

AB-04 female TTTACATAGTTTACTWATATGTTTCACACAGTTTGATAGCTTAGCTTTGTTCACTTTACT

AB-05 female TTTACATAGTTTACTAATATGTTTCACACAGTTTGATAGCTTAGCTTTGTTCACTTTACT

AB-06 female TTTACATAGTTTACTWATATGTTTCACACAGTTTGATAGCTTAGCTTTGTTCACTTTACT

AB-07 female TTTACATAGTTTACTWATATGTTTCACACAGTTTGATAGCTTAGCTTTGTTCACTTTACT

AB-08 female TTTACATAGTTTACTWATATGTTTCACACAGTTTGATAGCTTAGCTTTGTTCACTTTACT

AB-09 female TTTACATAGTTTACTWATATGTTTCACACAGTTTGATAGCTTAGCTTTGTTCACTTTACT

AB-10 female TTTACATAGTTTACTWATATGTTTCACACAGTTTGATAGCTTAGCTTTGTTCACTTTACT

AB-11 female TTTACATAGTTTACTWATATGTTTCACACAGTTTGATAGCTTAGCTTTGTTCACTTTACT

AB-12 female TTTACATAGTTTACTAATATGTTTCACACAGTTTGATAGCTTAGCTTTGTTCACTTTACT

AB-13 male TTTACATAGTTTACTAATATGTTTCACACAGTTTGATAGCTTAGCTTTGTTCACTTTACT

AB-14 male TTTACATAGTTTACTWATATGTTTCACACAGTTTGATAGCTTAGCTTTGTTCACTTTACT

AB-15 male TTTACATAGTTTACTAATATGTTTCACACAGTTTGATAGCTTAGCTTTGTTCACTTTACT

AB-16 male TTTACATAGTTTACTAATATGTTTCACACAGTTTGATAGCTTAGCTTTGTTCACTTTACT

AB-17 male TTTACATAGTTTACTWATATGTTTCACACAGTTTGATAGCTTAGCTTTGTTCACTTTACT

AB-18 male TTTACATAGTTTACTWATATGTTTCACACAGTTTGATAGCTTAGCTTTGTTCACTTTACT

AB-19 male TTTACATAGTTTACTWATATGTTTCACACAGTTTGATAGCTTAGCTTTGTTCACTTTACT

AB-20 male TTTACATAGTTTACTAATATGTTTCACACAGTTTGATAGCTTAGCTTTGTTCACTTTACT

AB-21 male TTTACATAGTTTACTWATATGTTTCACACAGTTTGATAGCTTAGCTTTGTTCACTTTACT

AB-22 male TTTACATAGTTTACTAATATGTTTCACACAGTTTGATAGCTTAGCTTTGTTCACTTTACT

AB-23 male TTTACATAGTTTACTAATATGTTTCACACAGTTTGATAGCTTAGCTTTGTTCACTTTACT

AB-24 male TTTACATAGTTTACTAATATGTTTCACACAGTTTGATAGCTTAGCTTTGTTCACTTTACT

1 10 20 30 40 50 60

| | | | | | |

ss184964140

AB-01 female GCTACAGATTTTAAAGGTTAAAATGATTTACTTCCACTATCTAGAAGAACACTATTGAAA

AB-02 female GCTRCAGATTTTAAAGGTTAAAATGATTTACTTCCACTATCTAGAAGAACACTATTGAAA

AB-03 female GCTRCAGATTTTAAAGGTTAAAATGATTTACTTCCACTATCTAGAAGAACACTATTGAAA

AB-04 female GCTRCAGATTTTAAAGGTTAAAATGATTTACTTCCACTATCTAGAAGAACACTATTGAAA

AB-05 female GCTACAGATTTTAAAGGTTAAAATGATTTACTTCCACTATCTAGAAGAACACTATTGAAA

AB-06 female GCTRCAGATTTTAAAGGTTAAAATGATTTACTTCCACTATCTAGAAGAACACTATTGAAA

AB-07 female GCTRCAGATTTTAAAGGTTAAAATGATTTACTTCCACTATCTAGAAGAACACTATTGAAA

AB-08 female GCTRCAGATTTTAAAGGTTAAAATGATTTACTTCCACTATCTAGAAGAACACTATTGAAA

AB-09 female GCTRCAGATTTTAAAGGTTAAAATGATTTACTTCCACTATCTAGAAGAACACTATTGAAA

AB-10 female GCTRCAGATTTTAAAGGTTAAAATGATTTACTTCCACTATCTAGAAGAACACTATTGAAA

AB-11 female GCTRCAGATTTTAAAGGTTAAAATGATTTACTTCCACTATCTAGAAGAACACTATTGAAA

AB-12 female GCTACAGATTTTAAAGGTTAAAATGATTTACTTCCACTATCTAGAAGAACACTATTGAAA

AB-13 male GCTACAGATTTTAAAGGTTAAAATGATTTACTTCCACTATCTAGAAGAACACTATTGAAA

AB-14 male GCTRCAGATTTTAAAGGTTAAAATGATTTACTTCCACTATCTAGAAGAACACTATTGAAA

AB-15 male GCTACAGATTTTAAAGGTTAAAATGATTTACTTCCACTATCTAGAAGAACACTATTGAAA

AB-16 male GCTACAGATTTTAAAGGTTAAAATGATTTACTTCCACTATCTAGAAGAACACTATTGAAA

AB-17 male GCTRCAGATTTTAAAGGTTAAAATGATTTACTTCCACTATCTAGAAGAACACTATTGAAA

AB-18 male GCTRCAGATTTTAAAGGTTAAAATGATTTACTTCCACTATCTAGAAGAACACTATTGAAA

AB-19 male GCTRCAGATTTTAAAGGTTAAAATGATTTACTTCCACTATCTAGAAGAACACTATTGAAA

AB-20 male GCTACAGATTTTAAAGGTTAAAATGATTTACTTCCACTATCTAGAAGAACACTATTGAAA

AB-21 male GCTRCAGATTTTAAAGGTTAAAATGATTTACTTCCACTATCTAGAAGAACACTATTGAAA

AB-22 male GCTACAGATTTTAAAGGTTAAAATGATTTACTTCCACTATCTAGAAGAACACTATTGAAA

AB-23 male GCTACAGATTTTAAAGGTTAAAATGATTTACTTCCACTATCTAGAAGAACACTATTGAAA

AB-24 male GCTACAGATTTTAAAGGTTAAAATGATTTACTTCCACTATCTAGAAGAACACTATTGAAA

AB-01 female GAAATGGCAGAGCAGAACGATTTACTGTCGCACATCAGTTGAAGCTCAATATTGCTGTCA

AB-02 female GAAATGGCAGAGCAGAACGATTTACTGTCGCACATCAGTTGAAGCTCAATATTGCTGTCA

AB-03 female GAAATGGCAGAGCAGAACGATTTACTGTCGCACATCAGTTGAAGCTCAATATTGCTGTCA

AB-04 female GAAATGGCAGAGCAGAACGATTTACTGTCGCACATCAGTTGAAGCTCAATATTGCTGTCA

AB-05 female GAAATGGCAGAGCAGAACGATTTACTGTCGCACATCAGTTGAAGCTCAATATTGCTGTCA

AB-06 female GAAATGGCAGAGCAGAACGATTTACTGTCGCACATCAGTTGAAGCTCAATATTGCTGTCA

AB-07 female GAAATGGCAGAGCAGAACGATTTACTGTCGCACATCAGTTGAAGCTCAATATTGCTGTCA

AB-08 female GAAATGGCAGAGCAGAACGATTTACTGTCGCACATCAGTTGAAGCTCAATATTGCTGTCA

AB-09 female GAAATGGCAGAGCAGAACGATTTACTGTCGCACATCAGTTGAAGCTCAATATTGCTGTCA

AB-10 female GAAATGGCAGAGCAGAACGATTTACTGTCGCACATCAGTTGAAGCTCAATATTGCTGTCA

AB-11 female GAAATGGCAGAGCAGAACGATTTACTGTCGCACATCAGTTGAAGCTCAATATTGCTGTCA

AB-12 female GAAATGGCAGAGCAGAACGATTTACTGTCGCACATCAGTTGAAGCTCAATATTGCTGTCA

AB-13 male GAAATGGCAGAGCAGAACGATTTACTGTCGCACATCAGTTGAAGCTCAATATTGCTGTCA

AB-14 male GAAATGGCAGAGCAGAACGATTTACTGTCGCACATCAGTTGAAGCTCAATATTGCTGTCA

AB-15 male GAAATGGCAGAGCAGAACGATTTACTGTCGCACATCAGTTGAAGCTCAATATTGCTGTCA

AB-16 male GAAATGGCAGAGCAGAACGATTTACTGTCGCACATCAGTTGAAGCTCAATATTGCTGTCA

AB-17 male GAAATGGCAGAGCAGAACGATTTACTGTCGCACATCAGTTGAAGCTCAATATTGCTGTCA

AB-18 male GAAATGGCAGAGCAGAACGATTTACTGTCGCACATCAGTTGAAGCTCAATATTGCTGTCA

AB-19 male GAAATGGCAGAGCAGAACGATTTACTGTCGCACATCAGTTGAAGCTCAATATTGCTGTCA

AB-20 male GAAATGGCAGAGCAGAACGATTTACTGTCGCACATCAGTTGAAGCTCAATATTGCTGTCA

AB-21 male GAAATGGCAGAGCAGAACGATTTACTGTCGCACATCAGTTGAAGCTCAATATTGCTGTCA

AB-22 male GAAATGGCAGAGCAGAACGATTTACTGTCGCACATCAGTTGAAGCTCAATATTGCTGTCA

AB-23 male GAAATGGCAGAGCAGAACGATTTACTGTCGCACATCAGTTGAAGCTCAATATTGCTGTCA

AB-24 male GAAATGGCAGAGCAGAACGATTTACTGTCGCACATCAGTTGAAGCTCAATATTGCTGTCA

1 10 20 30 40 50 60

| | | | | | |

AB-01 female TTGGCCTAATGGATAATCTAACTTCCTT

AB-02 female TTGGCCTAATGGATAATCTAACTTCCTT

AB-03 female TTGGCCTAATGGATAATCTAACTTCCTT

AB-04 female TTGGCCTAATGGATAATCTAACTTCCTT

AB-05 female TTGGCCTAATGGATAATCTAACTTCCTT

AB-06 female TTGGCCTAATGGATAATCTAACTTCCTT

AB-07 female TTGGCCTAATGGATAATCTAACTTCCTT

AB-08 female TTGGCCTAATGGATAATCTAACTTCCTT

AB-09 female TTGGCCTAATGGATAATCTAACTTCCTT

AB-10 female TTGGCCTAATGGATAATCTAACTTCCTT

AB-11 female TTGGCCTAATGGATAATCTAACTTCCTT

AB-12 female TTGGCCTAATGGATAATCTAACTTCCTT

AB-13 male TTGGCCTAATGGATAATCTAACTTCCTT

AB-14 male TTGGCCTAATGGATAATCTAACTTCCTT

AB-15 male TTGGCCTAATGGATAATCTAACTTCCTT

AB-16 male TTGGCCTAATGGATAATCTAACTTCCTT

AB-17 male TTGGCCTAATGGATAATCTAACTTCCTT

AB-18 male TTGGCCTAATGGATAATCTAACTTCCTT

AB-19 male TTGGCCTAATGGATAATCTAACTTCCTT

AB-20 male TTGGCCTAATGGATAATCTAACTTCCTT

AB-21 male TTGGCCTAATGGATAATCTAACTTCCTT

AB-22 male TTGGCCTAATGGATAATCTAACTTCCTT

AB-23 male TTGGCCTAATGGATAATCTAACTTCCTT

AB-24 male TTGGCCTAATGGATAATCTAACTTCCTT
